# An accurate DFT study within conformational survey of the d-form serine−alanine protected dipeptide

**DOI:** 10.1186/s13065-023-01051-9

**Published:** 2023-10-13

**Authors:** Behzad Chahkandi, Mohammad Chahkandi

**Affiliations:** 1 Department of Chemistry, Mashhad Branch, Islamic Azad University, Mashhad, Iran; 2 Department of Chemistry, Hakim Sabzevari University, Sabzevar, 96179-76487 Iran

**Keywords:** For‒d‒Ser‒ d‒Ala‒NH2 dipeptide, Ramachandran map, *β*–turn, DFT, QTAIM, PCM

## Abstract

The conformational analysis of n-formyl-d-serine-d-alanine-NH_2_ dipeptide was studied using density functional theory methods at B3LYP, B3LYP‒D3, and M06‒2X levels using 6‒311 + G (*d,p*) basis set in the gas and water phases. 87 conformers of 243 stable ones were located and the rest of them were migrated to the more stable geometries. Migration pattern suggests the more stable dipeptide model bears serine in* β*_*L*_*, γ*_*D*_*, γ*_*L*_ and the alanine in* γ*_*L*_ and* γ*_*D*_ configurations. The investigation of side‒chain‒backbone interactions revealed that the most stable conformer,* γ*_*D*_^*–*^*γ*_*L*_, is in the *β*‒turn region of Ramachandran map; therefore, serine-alanine dipeptide model should be adopted with a *β*‒turn conformation. Intramolecular hydrogen bonding in *β*‒turns consideration by QTAIM disclosed *γ*_*D*_^*–*^*γ*_*L*_ includes three hydrogen bonds. The computed UV‒Vis spectrum alongside of NBO calculation showed the five main electronic transition bands derived of n → n^*^ of intra‒ligand alanine moiety of dipeptide structure.

**Supplementary Information:**

The online version contains supplementary material available at 10.1186/s13065-023-01051-9.

## Introduction

The functional properties of the proteins and peptides constructing the living bodies depend on their three‒dimensional (3D) structure. The conformational properties of these bio‒ingredients are directly impressed by their functional structures. Besides, the electronic and vibrational characters of a molecule are connected to its conformational structure. Amino acids, as the building blocks of peptides and proteins [1], are some of the designator biochemical regulators, such as neurotransmitters [2, 3] and autophagy regulators [4–6]. Except of glycine, the rest of whole 20 amino acids have both d‒ and l‒ enantiomeric forms with different physiological and biological functions. The optical purity have key effect on property and should be carefully controlled, i.e. l‒amino acids, in most cases, show a biocompatible identity, but d ‒amino acids may have harmless effects [7]. For a long time, it was thought which d‒amino acids are abnormal and absent in mammals. Nevertheless, recent studies approved the presence of d‒amino acids, such as d‒aspartate, d‒serine (ser), and d‒alanine (ala), in mammals [8, 9]. Additionally, enzymatic studies have elucidated that the synthesis and metabolism of d‒amino acids are physiologically regulated [10–13]. Furthermore, a reliable evidence emphasizes the role of d‒amino acids in the development, pathophysiology, and cancer therapy [14].

The ser‒ala dipeptide is constructed by coupling two contrary amino acids: the former is a polar/hydrophilic (ser) and the latter is a non‒polar/hydrophobic (ala) kind. Ser and ala bear hydroxylic and aliphatic side‒chains of methyl and hydroxymethyl, respectively. These specific properties of those unparalleled ala and ser residues give them some critical functional roles in many bio‒organisms. For example, d‒ser has an essential task in the central nervous system (CNS) of rodents and humans, including of midbrain, cerebellum, and spinal cord [15–18]. According to the secondary structure of proteins and peptides, the functional properties of them have traditionally visualized, appropriated, and analyzed.

d‒amino acids can be regarded as the constituents of bio‒stable polypeptides or some bio‒based materials. The stereo‒specificity of proteases can be pursued and controlled in polymerization reactions [19]. The protein structure and folding can be decoded by conformational space mapping of small peptide fragments. As noted above, incorporating d‒enantiomers of all amino acids within a peptide sequence would allow the synthetization and preparation of the effective drugs. For example, leuprolide acetate known as a cancer drug, and penicillin consist of d‒leucine‒l‒leucine and d‒ala‒ d‒ala, respectively [20, 21]. The results of conformational studies of the MeCO‒ala‒ala‒NH‒Me protected dipeptide about its four different configurations (l‒ala‒l‒ala, d‒ala‒ d‒ala, l‒ala‒ d‒ala, and d‒ala‒ d‒ala), showed that the most stable one would be $\beta_{L} \gamma_{L}^{{}}$, $\beta_{L} \gamma_{D}^{{}}$, $\beta_{L} \gamma_{D}^{{}}$, and $\beta_{L} \gamma_{L}^{{}}$, respectively [22]. Moreover, the topologies of conformational potential energy surfaces of d‒ and l‒alanyl residues on a glycine unit of n‒Ac‒ala‒gly‒ala‒NHMe [23] and n‒Ac‒ala‒gly[β]‒ala‒NHMe [24] tripeptides were investigated.

The thermodynamic properties of protolytic equilibria of d/l‒alanyl‒d/l‒serine dipeptide have been determined in aqueous solutions by means of potentiometry and calorimetry [25]. For the first time, herein, the complete conformational analysis of HCO‒ser‒ala‒NH_2_ dipeptide were investigated by the aid of quantum chemical calculations. All peptide bonds of HCO‒ser‒ala‒NH_2_ dipeptide are in the trans isomeric state, and chiral *α*‒carbons are in the D enantiomeric state [26].

N‒formyl and amine functionalities were attached to the terminus of dipeptides. These moieties safeguard the mimetic of conformational properties of polypeptides and protein segments and simulate the spatial and inductive contacts of those adjacent ser‒ala (sa) amino acid residues.

**Scheme 1:**
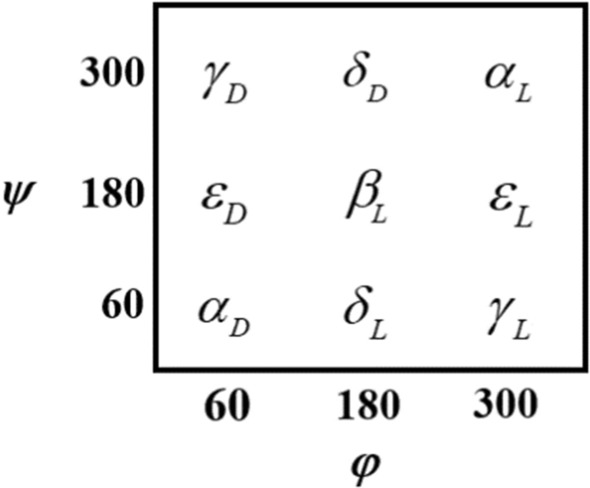
Conformational nomenclature peptide conformers on the Ramachandran map for a peptide (PCO–NH–CHR–CO–NHQ, P and Q may be H or CH_3_)

The 243 possible different conformers of HCO‒d‒ser‒d‒ala‒NH_2_ on the Ramachandran map, [27, 28] were investigated at the B3LYP/6‒311 + G *(d,p*) level of theory. However, it is possible that some conformers were lost and less than 243 conformers would be found. Migrations of nonexistent conformers can assist to identification of the relative stabilities and instabilities of the different conformations (ref Scheme 1).

**Fig. 1:**
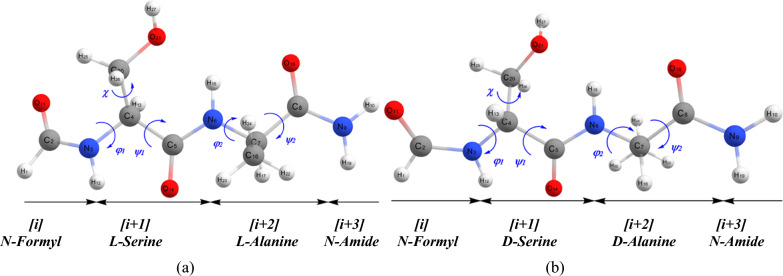
Schematic of the standardized numbering system, applied to the conformational structure of diamides of HCO‒L‒ser‒L‒ala‒NH_2_ (**a**) and HCO‒D‒ser‒D‒ala‒NH_2_ (**b**) protected dipeptide, showing all BB torsional angles. The four sections of the structure of ser‒ala dipeptide are: N‒terminus protecting group (For=HCO), ser residue, ala residue, and the NH_2_ group (ϕ_1_=C_2_‒N_3_‒C_4_‒C_5_, ψ_1_=N_3_‒C_4_‒C_5_‒N_6_, ϕ_2_=C_5_‒N_6_‒C_7_‒C_8_, ψ_2_=N_6_‒C_7_‒C_8_‒N_9_, χ=N_3_‒C_4_‒C_20_‒N_21_)

The numbering of atomic sequence in the sa‒protected dipeptide was performed under the standardized numbering system [29], which provides the necessary modeling data for the further studies of longer or shorter peptides (Fig. 1). Moreover, the *β*‒turn conformations of HCO‒d‒ser‒d‒ala‒NH_2_ protected dipeptide were also investigated by the B3LYP‒D3/6‒311 + G (*d,p*) and M06‒2x/6‒311 + G (*d,p*) levels of theory in the gas and water solvent. The L and D forms of ser-ala protected dipeptide have been investigated in the previous [26], and present studies, respectively that are depicted in Fig. 1.

The backbone (BB) conformation of the *β*‒turn is highly variable that can be classified based on the dihedral angles contents of ϕ and ψ of the central residue (Table 1). The deviation of ± 30° from these canonical extents is allowed within these foresaid angles, whereas the fourth ones can be deviated by ± 45° [30, 31].

**Table 1:** Torsional definitions of *β*‒turns by their torsional angles,$\varphi_{i + 1}$, $\psi_{i + 1}$,$\varphi_{i + 2}$ and $\psi_{i + 2}$

$$\beta - turn\begin{array}{*{20}c} {type} \\ \end{array}$$	BB torsional angle values*			
	$\varphi_{i + 1}$	$\psi_{i + 1}$	$\varphi_{i + 2}$	$\psi_{i + 2}$
$I$	− 60	− 30	− 90	0
$I^{\prime}$	60	30	90	0
$II$	− 60	120	80	0
$II^{\prime}$	60	− 120	− 80	0
$III$	− 60	− 30	− 60	− 30
$III^{\prime}$	60	30	60	30
$IV$	− 61	10	− 53	17
$V$	− 80	80	80	− 80
$V^{\prime}$	80	− 80	− 80	80
$VIa1$	− 60	120	− 90	0
$VIa2$	− 120	120	− 60	0
$VIb$	− 135	135	− 75	160
$VIII$	− 60	− 30	− 120	120

^*^The three torsional angles varying by ± 30° and with one angle allowed to deviate by 45° [30, 31]

**Scheme 2:**
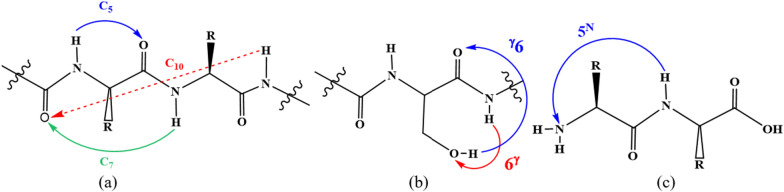
The intramolecular interactions and corresponding notation according to the H‒Bonding patterns: **a** BB‒BB interactions **b** SC‒BB interactions **c** N‒terminus interaction

Usually, *β*‒turn secondary structures exist in types I, II, and III. However, their conformational enantiomers (I′, II′, III′), and VIa1, VIa2, VIb, and VIII types of *β*‒turns are less common. Conventionally, eight types of *β*‒turns have been distinguished and those not defined are classified as type IV. The turns comprise *n* consecutive residues (*i* to *i* + *n*), according to some reports, the distance between α‒carbon of residues symbolized *as i* and *i* + *n*, must be smaller than 7 Å or 7.5 Å [32, 33]. The turns are composed of *γ*‒turns (*n* = 3), [34] *β*‒turns (*n* = 4), [35] *α*‒turns (*n* = 5) [36, 37] and *π*‒turns (*n* = 6) [38, 39]. The participated amino acids in the case of noted turns form 7 (C_7_), 10 (C_10_), 13 (C_13_) and 16 (C_16_) membered hydrogen bonded (H‒bonded) rings for *γ*‒*, β*‒*, α*‒ and* π*‒turns, respectively. The term Cn interaction is used when the H‒bond (HB) leads to the formation of a n‒membered ring, include of the donating NH and the accepting CO groups [40]. The most *β*‒turn structures, as defined by C.M. Venkatachalam were characterized by a HB between the N‒H and C=O of residues *i* and *i* + 3, respectively [41], that generates a 10 membered ring. The C_n_ interactions that lead to the formation of a n‒membered ring for n = 5, 7, and 10 were shown in Scheme 2. While the folding of a peptide into a *β*‒turn is a multifaceted process driven in part by local torsional preferences, inter‒strand H‒bonding can offer additional stabilization of the turn [42]. Another nomenclature is needed when SCs or the N and/or C terminus are involved in H‒bonding [43–49], in the first case the size of formed ring is denoted with ″n″, and the position of side chain group related to its α‒C atom is given by Greek letters (e.g., γ, δ, ε) as superscript (left‒hand side if it’s in a donating function, right‒hand side for an acceptor function) [47, 48]. In addition, the N‒ or C‒terminus participated in a HB is marked with ″N″ or ″C″ as superscripted to the number ″n″ of the ring size. The superscript is placed on the left and right‒hand side of ″n″, if the terminus acts as a HB donor and acceptor, respectively (see Fig. 1 and Scheme 2).

The further structural consideration of the studied stable conformers of sa‒protected dipeptide was applied by the UV‒Vis spectrum and Frontier Molecular Orbitals (FMOs) investigations. These complementary results can be used for better understanding of the dipeptide structure.

In the present research, the new calculations including of the effect of solvent (water) dielectric constant on the stability of *β*−turn conformers and Frontier Molecular Orbitals investigations by UV–Vis spectrum and NBO considerations of the most stable conformer of dipeptide model were performed. These interesting studies can be presented as the main novelty and differentiation related to the previous reported results by us [26]. Moreover, the non-covalent interactions (like HB) consideration was studied by quantum theory of atoms in molecules (QTAIM) and B3LYP‒D3 theoretical levels with good consistency that both results confirm each other.

## Computational methods

First, n−formyl−d−serine−d−alanine−NH_2_ was modeled with each residue and protecting group segments were separately for the entirety of the peptide chain numbered. Then, formyl and amide functionalities were attached to the N‒ and C‒terminuses of the dipeptide, respectively for safe guarding the mimetic of steric effects of the neighboring amino acid residues.

The sa‒protected dipeptide was separated into four units: (1) The N‒terminus protecting group, (2) the D‒ser residue, (3) the D‒ala residue, and (4) the C‒terminus protecting group. A standardized numbering system was separately used into and along the peptide BB to define the numbered dipeptide (Fig. 1). Therefore, every of the comprised unit can be shortened at any time for modeling the structure of a larger polypeptide [30]. The sa‒protected dipeptide conformation is characterized by the dihedral angles along the *α*‒carbon atom of the amino acid subgroups denoted as $\phi$**, **$\psi$**,** and $\chi$ [30, 31]. The *φ*, and *ψ* dihedral angles specify the BB, while the χ describes the side-chain conformation of the dipeptide. The rotations around $N - C_{\alpha }$, $C_{\alpha } - CO$, and $OC - NH$(i.e., the peptide bond) bonds are specified by φ, ψ, and ω dihedral angles, respectively. Five dihedral angles *φ*_*1*_*, ψ*_*1*_*, φ*_*2*_*, ψ*_*2*_, and *χ* were considered most relevant to the shape and stability of sa-protected dipeptide model. The rotation around χ resulted in side-chain conformers that are defined as gauche ( +) (g ^+^), anti (a), and gauche ( −) (g^−^). Initially, the fully relaxed optimization in the gas phase without any symmetry constraints (C1 symmetry for all conformations assumed) was employed at B3LYP [50, 51] and M06‒2X [52] levels of theory using the 6‒311 + G (*d,p*) basis set. M06‒2X is a hybrid meta exchange‒correlation functional and one of the best functioning for studying the organic and biological small molecules [53]. In order to estimate the effect of dispersion interactions, the dispersion energy corrected B3LYP‒D3 functional [51] is also used here to optimize the structure and calculate frequencies of *β*‒turn conformers. Additionally, the effect of solvent on *β* − turn structures was calculated using the SCRF keyword with Tomasi’s polarized continuum model (PCM; *ε* = 78*.*36 for water) [54, 55]. Frequency calculations at the same levels of theory were also performed to characterize the stationary points as local minima on the potential energy surface and to evaluate the zero‒point vibrational energy (ZPE). The absence of imaginary frequency on the calculated vibrational spectrum confirms that the structure corresponds to the minimum energy. The different conformations of the dipeptide HCO−d−ser−d−ala−NH_2_ were optimized by restraining the serine residues to the nine conformations, ($\alpha_{D} ,\alpha_{L} ,\varepsilon_{D} ,\varepsilon_{L} ,\gamma_{D} ,\gamma_{L} ,\delta_{D} ,\delta_{L} ,\beta_{L}$), on the Ramachandran map (Scheme 1) and varying the alanine residues for each of the nine optimized conformations. Since it can be expected that three minima (g + , a, g^−^) along χ side − chain dihedral angle are present, the multidimensional conformational analysis (MDCA) would lead to the existence of 3^5^ = 243 conformers. In addition, the theoretical electronic transitions (ETs) of vacuous phase of UV‒Vis spectrum of dipeptide optimized geometry were done using time‒dependent DFT (TD‒DFT) calculations. Both of singlet and triplet states were respected; while excitations to triplet excited states were prohibited. Partial atomic charges and Natural bond orbital (NBO) analyses were also computed using B3LYP‒D3/6‒311 + G (*d,p*) level of theory. All computations concerning conformers of serine‒alanine protected dipeptide were carried out by the GAUSSIAN 09 program package, [56] at 298.15 K and 1.0 atm. Finally, the computations of the QTAIM [57] were performed for analyzing the nature of the intramolecular hydrogen bonds (IHBs) of *β* − turn structures. The wave functions used in the QTAIM analyses were generated at the B3LYP‒D3/6‒311 + G (*d,p*) and M06‒2X/6‒311 + G (*d,p*) levels of theory. The related calculations were carried out using the AIMAll program [58].

## Results and discussion

### Side-chain conformational study

The conformational structure of diamides of sa‒protected dipeptide and its standard numbering system is depicted in Fig. 1. The three different stable serine side‒chain conformers where associated with every BB conformation was obtained by varying side‒chain dihedral angle, χ, at 30º intervals from 0 to 360º. Results for the relative energies (kcal mol^−1^) of different conformations bearing different side‒chain dihedral angles ($\chi$) are summarized in Table 2.

**Table 2:** Calculated (B3LYP/6‒311 + G (*d,p*)) relative energies, $\Delta E_{rel}$(kcal mol^−1^) of different side‒chain conformers of HCO‒D‒ser‒D‒ala‒NH_2_

$\chi$	0	30	**60**	90	120	150	**180**	210	240	270	**300**	330	360
$\Delta E_{rel}$	2.44	7.24	**5.07**	6.29	6.46	1.07	**0.00**	3.40	5.99	3.92	**2.48**	5.90	2.44

The bold values correspond to the three different stable serine side-chain conformers, including gauche (+) ($\chi$ = + 60°), anti ($\chi$ = 180°), and gauche (−) ($\chi$ = − 60°), respectively

The anti ($\chi$ = 180º) conformer is the most stable and the relative energies of gauche ( +) ($\chi$ =  + 60º) and gauche ( −) ($\chi$ =  − 60º) conformers are 2.48 and 5.07, respectively.

The D‒ and L‒ enantiomers are mirror image of each other. The results of our previous work on the L form of sa dipeptide showed that gauche ( +) conformer is more stable than gauche ( −). This is also in agreement with the results that the anti-conformer is the most stable [26].

### Backbone conformational analysis

The three dihedral angles *φ*, *ψ*, and *ω*, lead to a potential energy hypersurface (PEHS; *E* = *E (φ,ψ,ω)*). Since in a trans amide bond conformer, the *ω* torsional angle has a constant value of 180º, the hypersurface *E* = *E (φ,ψ,ω)* can be simplified to *E* = *E (φ,ψ)*. The calculated results at the B3LYP/6‒311 + G *(d,p*) show that 87 stable conformers were characterized, and the rest changed to the more stable ones. Initial and final optimized conformations (including anti, gauche ( +), and gauche ( −) side-chain forms) of all 243 possible ones of the sa-protected dipeptide were reported in supplementary information (Additional file 1: Table S1). The conformations of 35 models of both ser and ala residues have migrated to the altered conformations (red and * marked ones in Additional file 1: Table S1). In contrast, within the 121 instances, only one of them has migrated, and another one remained unchanged (blue ones in Additional file 1: Table S1).

#### Migration patterns of alanine residue

**Fig. 2:**
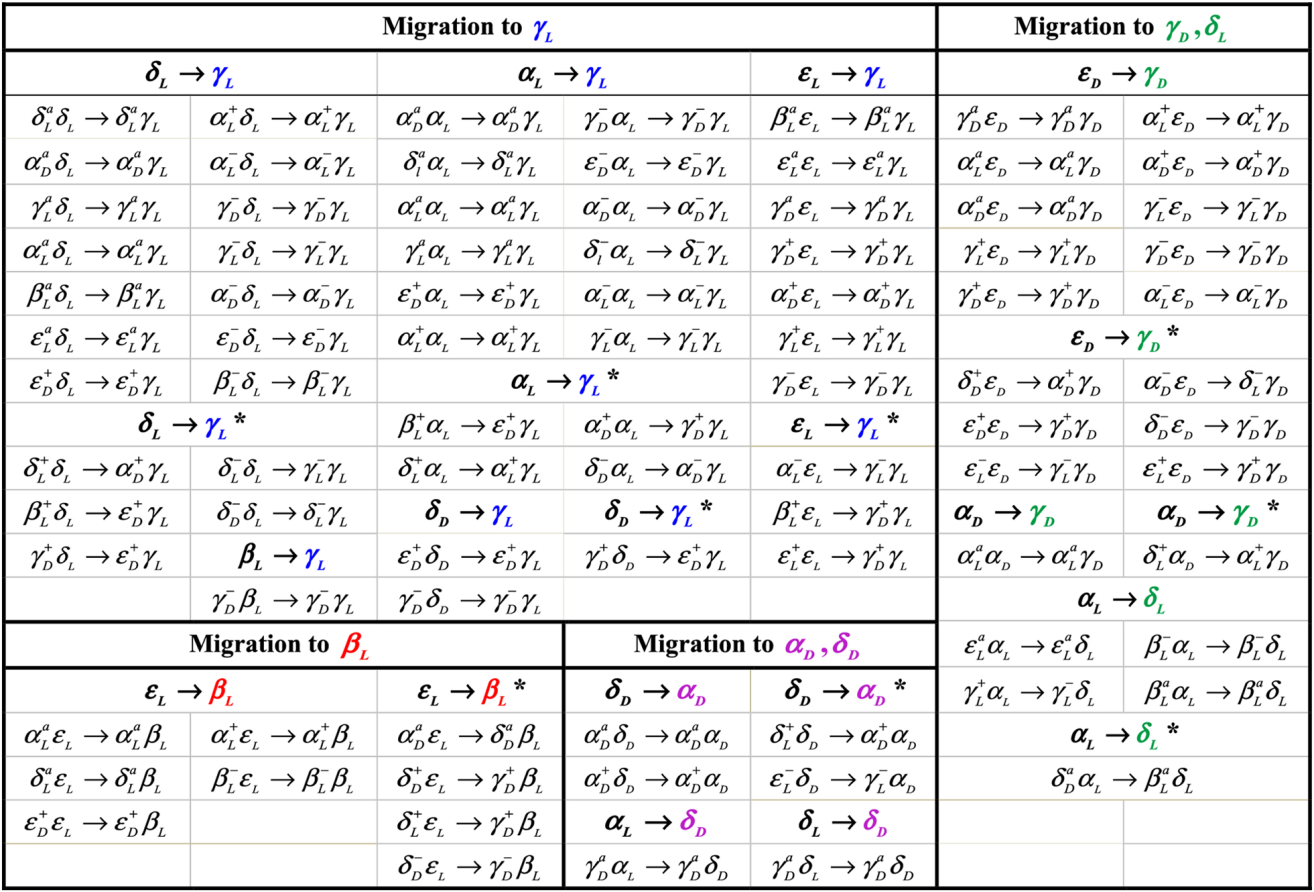
Migration patterns of the ala residue of the For‒D‒ser‒D‒ala − NH_2_ dipeptide at the B3LYP/6‒311 + G *(d,p*) level of theory

The migration patterns of alanine residue of sa‒protected dipeptide are shown in Fig. 2. The obtained results indicate that in 36 cases of 49 conformers bearing migration of ala BB to $\gamma_{L}$,the ser residue retained its original conformation, while, in about 13 instances, the ser residue also changed to a different conformation. Among the 36 cases mentioned above, in 12, 14, 7, 2, and 1 models, ala BB conformers changed from $\alpha_{L}$, $\delta_{L}$, $\varepsilon_{L}$, $\delta_{D}^{{}}$ and $\beta_{L}$ to $\gamma_{L}$, respectively, and in 13 cases, ala BB conformers changed from $\delta_{L}$, $\alpha_{L}$, $\delta_{D}^{{}}$, and $\varepsilon_{L}$ to $\gamma_{L}$, within 5, 4, 1, and in 3 instances, respectively. Moreover, in 18 cases, the ala BB conformers migrated from $\varepsilon_{D}$ and $\alpha_{D}$ to $\gamma_{D}$. While, 11 instances preserved the original conformation of ser residue, and in 7 ones, the ser residue changed to different conformations. In 10 models of the all 11 mentioned cases, ala BB conformers changed from $\varepsilon_{D}$ to $\gamma_{D}$ and from $\alpha_{D}$ to $\gamma_{D}$ in 1 model. Additionally in 7 cases, ala BB conformers changed from $\varepsilon_{D}$ and $\alpha_{D}$ to $\gamma_{D}$, within 6 and 1 instances, respectively.

According to the results shown in Fig. 2, the ala residue of $\varepsilon_{L}$,$\alpha_{L}$ and $\delta_{D}^{{}}$ BB conformers migrated to $\beta_{L}$,$\delta_{L}$, and $\alpha_{D}$, within 9, 5, and 4 instances, respectively. Meanwhile, among of 18 conformers described above, in 5, 4, and 2 cases, the ser residue retained its initial conformational structures, but in 4, 1, and 2 ones, the ser residue also changed to a different conformation, respectively.

Finally, in 2 instances, whereas the ser residue retained its original conformation, the ala residue also changed from $\alpha_{L}$ and $\delta_{L}$ to $\delta_{D}^{{}}$ conformation. In summary, from all 87 cases bearing the dipeptide migration, in 60 conformers, the serine residue retained its original structure while, changed to a different form within the rest of the 27 cases. Our results suggest that the $\beta_{L}$,$\delta_{L}$,$\alpha_{D}$, and $\delta_{D}^{{}}$ conformers of Ala destabilize the model, while greater stability is conferred by the $\gamma_{L}$ and $\gamma_{D}$ conformers.

#### Migration patterns of serine residue

**Fig. 3:**
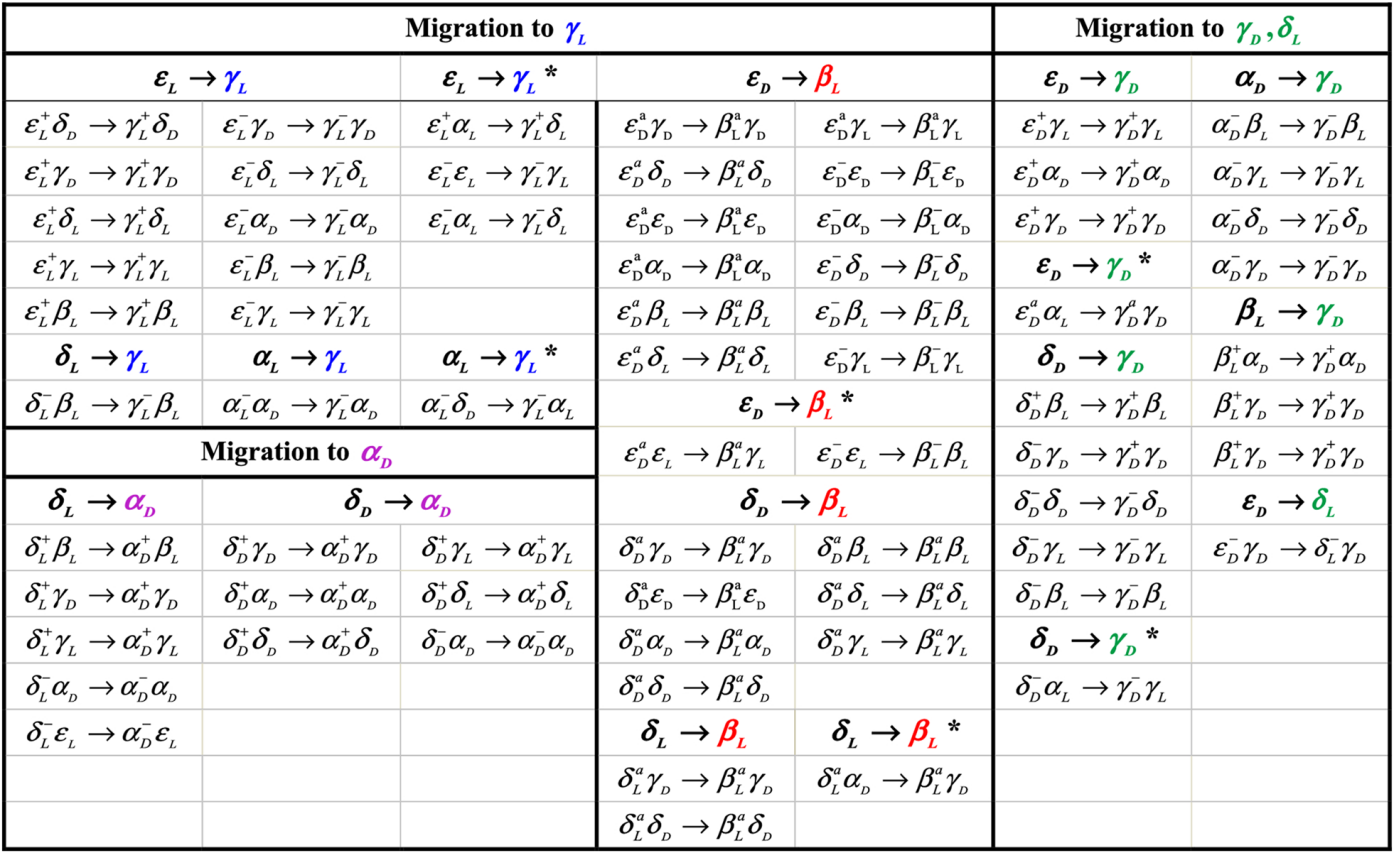
Migration patterns of the ser residue of the For‒D‒ser‒D‒ala − NH_2_ dipeptide at the B3LYP/6‒311 + G *(d,p*) level of theory

The migration patterns for the serine residue in the sa-protected dipeptide are displayed in Fig. 3. The optimized geometries of different conformations of sa‒protected dipeptide show that in the 23 instances, the ser BB conformers migrated from $\varepsilon_{D}$, $\delta_{D}^{{}}$, and $\delta_{L}$ to $\beta_{L}$, that in the 21 cases (12, 7, and 2 items migrate from $\varepsilon_{D}$ to $\delta_{D}^{{}}$, and $\delta_{L}$ to $\beta_{L}$, respectively), the ala residue retained its conformation and, in 3 cases, changed to the different conformations. And also, in 39 instances, while the ala residue remained in its original conformation, the ser residue migrated to $\gamma_{D}$, $\gamma_{L}$, $\alpha_{D}$, and $\delta_{L}$ (15, 12, 11, and 1 items, respectively), but in 6 instances, the ser residue migrated to $\gamma_{L}$, and $\gamma_{D}$(4 and 2 items, respectively), whereas, the ala residue also converted to the different conformers (see Fig. 3).

The obtained results indicate that among the 156 migrated conformers of ser and ala residues of BB conformers of sa‒protected dipeptide, 65, 33, 35, 15, 6, and 2 cases converted to $\gamma_{L}$, $\beta_{L}$, $\gamma_{D}$, $\alpha_{D}$, $\delta_{L}$, and $\delta_{D}^{{}}$, respectively. According to these results, it can be suggested that $\alpha_{D}$ and $\delta_{L}$ conformers of ser residue cause the instability of the dipeptide while $\beta_{L}$, $\gamma_{D}$ and $\gamma_{L}$ conformers make a more stable model. Moreover, the $\beta_{L}$, $\delta_{L}$, $\alpha_{D}$, and $\delta_{D}^{{}}$ conformers within the ala residue causes the dipeptide to be unstable, inversely, $\gamma_{L}$ and $\gamma_{D}$ bring the more stability for the model. In the previous study about the conformational analysis of L enantiomer of sa‒protected dipeptide, it became evident that the $\beta_{L}$, $\gamma_{L}$, and $\gamma_{D}$ conformations within the ser residue and the $\gamma_{D}$ and $\gamma_{L}$ conformations of the ala residue create more stable dipeptide [26].

### *Energetic study of For*‒*d*‒*ser*‒*d-ala−NH*_*2*_* dipeptide*

The obtained computed relative and Gibbs free energies of all found conformations of sa‒dipeptide are shown in Table 3. The values of relative Gibbs free energy (∆G) are shown in the parentheses The relative energies were calculated by comparing the lowest energy conformer ($\gamma_{D}^{ - } \gamma_{L}$). Our results indicate that $\gamma_{D}^{ - } \gamma_{L}$ and $\delta_{L}^{ + } \varepsilon_{D}$ are the most stable and unstable conformations of For*−*d*−*ser*−*d*−*ala*−*NH_2_ dipeptide in the gas phase, respectively (see Table 3). The amounts of the relative and Gibbs free energies of the most unstable conformer, ($\delta_{L}^{ + } \varepsilon_{D}$), are calculated as 19.65 and 18.00 kcal mol^−1^, respectively. The conformations of For*−*d*−*ser*−*d*−*ala*−*NH_2_ dipeptide that have not been found and have migrated to a different conformation were symbolled with the N/F sign(s). The three more stable conformers of anti, gauche ( +), and gauche ( −) BB conformers of sa‒protected dipeptide are ($\gamma_{D}^{a} \gamma_{L}$, $\beta_{L}^{a} \gamma_{L}$, $\beta_{L}^{a} \varepsilon_{D}$), ($\gamma_{D}^{ + } \gamma_{L}$,$\gamma_{D}^{ + } \gamma_{D}^{{}}$,$\gamma_{D}^{ + } \alpha_{L}$), and ($\gamma_{D}^{ - } \gamma_{L}$,$\gamma_{D}^{ - } \gamma_{D}^{{}}$,$\gamma_{D}^{ - } \alpha_{D}$), with the energetic values of (3.25*(2.95),* 4.13*(3.14),* 4.29*(4.29)*), (5.91*(5.16),* 8.22*(7.20),* 8.27*(6.23)*) and (0.00*(0.00),* 2.30*(2.13),* 4.03*(3.88)*), respectively. These results confirm the previous section that for the ser and ala residues, the ($\beta_{L}$,$\gamma_{L}$,$\gamma_{D}$) and ($\gamma_{L}$,$\gamma_{D}$) conformers bring more stability to the sa‒protected dipeptide. The results of Table 3 indicate that among anti, gauche ( +), and gauche ( −), the ($\gamma_{D}^{a} \gamma_{L}$,$\alpha_{D}^{a} \gamma_{D}$), ($\gamma_{D}^{ + } \gamma_{L}$,$\delta_{L}^{ + } \varepsilon_{D}$), and ($\gamma_{D}^{ - } \gamma_{L}$,$\gamma_{L}^{ - } \beta_{L}$), BB conformers of sa‒protected dipeptide have the highest and lowest stability, respectively. The values of the relative and Gibbs free energies of $\alpha_{D}^{a} \gamma_{D}$, $\delta_{L}^{ + } \varepsilon_{D}$, and $\gamma_{L}^{ - } \beta_{L}$ are obtained as 16.91 (14.85), 19.65 (18.00), and 16.87 (14.27), respectively. Previous studies on L and D enantiomers of some mono peptides [59], have illustrated a distinct pattern between the two enantiomers; critical points that were missing from one monopeptide were clearly observed in the other, and vice versa. The d‒enantiomer was shown to be the mirror image of the l‒enantiomer. However, for the herein studied dipeptide, this issue was not clearly observed. According to the comparison of Ramachandran maps minima of l and k enantiomers of ser‒ala protected dipeptide it is obviously find that for example $\gamma_{L}^{a} \beta_{L}$ and $\gamma_{D}^{a} \beta_{L}$ conformations existed for For*−*d*−*ser*−*d*−*ala*−*NH_2_ while the same was true for $\gamma_{L}^{a} \beta_{L}$ conformation of For*−*d*−*ser*−*d*−*ala*−*NH_2_. When, the For*−*d*−*ser*−*d*−*ala*−*NH_2_ and For*−*d*−*ser*−*d*−*ala*−*NH_2_ dipeptides with a anti orientation of the ser side chain, had the alanine confined to $\delta_{L}^{{}}$ and the serine residues restrained to the nine conformations on the Ramachandran map, five found minima were present in l‒enantiomer dipeptide ($\beta_{L}^{a} \delta_{L}$, $\gamma_{L}^{a} \delta_{L}$, $\gamma_{D}^{a} \delta_{L}$, $\alpha_{D}^{a} \delta_{L}$, $\varepsilon_{D}^{a} \delta_{L}$), but are annihilated in D‒enantiomer (see Table 3 and ref. [26]).

**Table 3:** Calculated (B3LYP/6‒311 + G (*d,p*)) relative energies (kcal mol^−1^) of For‒D‒ser‒D‒ala‒NH_2_dipeptide in the gas phase

*BB[i] (ser)*	*BB[i* + *1] (ala)*								
	$\beta_{L}$	$\gamma_{L}$	$\gamma_{D}$	$\delta_{L}$	$\delta_{D}$	$\alpha_{L}$	$\alpha_{D}$	$\varepsilon_{L}$	$\varepsilon_{D}$
$\beta_{L}^{a}$	5.67*(3.92)*	**4.13*(3.14)***	6.33*(5.02)*	N/F	8.57*(7.15)*	N/F	8.00*(7.02)*	N/F	4.29*(4.29)*
$\gamma_{L}^{a}$	13.12*(9.97)*	9.67*(8.42)*	**12.30*(10.88)***	N/F	15.15*(13.56)*	N/F	15.22*(13.54)*	7.64*(7.34)*	15.17*(13.98)*
$\gamma_{D}^{a}$	6.02*(3.76)*	**3.25*(2.95)***	5.97*(5.26)*	N/F	8.67*(7.06)*	N/F	7.40*(6.89)*	N/F	N/F
$\delta_{L}^{a}$	14.01*(10.95)*	13.34*(11.05)*	N/F	N/F	N/F	N/F	N/F*	N/F	15.76*(14.34)*
$\delta_{D}^{a}$	N/F	N/F	N/F	N/F	N/F	N/F*	N/F	6.82*(5.16)*	N/F
$\alpha_{L}^{a}$	**6.97*(5.56)***	**6.72*(6.23)***	9.24*(8.45)*	N/F	13.03*(12.03)*	N/F	N/F	N/F	N/F
$\alpha_{D}^{a}$	15.48*(12.01)*	14.05*(12.08)*	**16.91*(14.85)***	N/F	N/F	N/F	**16.08*(14.60)***	N/F*	N/F
$\varepsilon_{L}^{a}$	10.04*(7.73)*	**8.38*(7.47)***	**10.51*(9.57)***	N/F	**14.53*(12.83)***	N/F	10.80*(10.69)*	N/F	10.86*(11.07)*
$\varepsilon_{D}^{a}$	N/F	N/F	N/F	N/F	N/F	N/F*	N/F	N/F*	N/F
$\beta_{L}^{ + }$	11.03*(7.72)*	N/F	N/F	N/F*	16.48*(13.31)*	N/F*	N/F	N/F	15.36*(12.91)*
$\gamma_{L}^{ + }$	12.07*(9.40)*	8.27*(7.12)*	**10.85*(9.57)***	11.36*(9.09)*	13.35*(11.58)*	N/F	13.18*(11.86)*	N/F	N/F
$\gamma_{D}^{ + }$	9.63*(7.41)*	**5.91*(5.16)***	8.22*(7.20)*	N/F*	N/F*	8.27*(6.23)*	10.25*(9.22)*	N/F	N/F
$\delta_{L}^{ + }$	N/F	N/F	N/F	N/F*	N/F*	N/F*	N/F*	N/F*	19.65*(18.00)*
$\delta_{D}^{ + }$	N/F	N/F	N/F	N/F	N/F	N/F*	N/F	N/F*	N/F*
$\alpha_{L}^{ + }$	**10.78*(8.74)***	**9.71*(8.59)***	11.97*(10.48)*	N/F	16.08*(14.40)*	N/F	14.74*(13.49)*	N/F	N/F
$\alpha_{D}^{ + }$	9.93*(7.42)*	8.50*(7.05)*	**11.49*(10.05)***	11.66*(9.33)*	N/F	N/F*	**9.80*(9.20)***	N/F	N/F
$\varepsilon_{L}^{ + }$	N/F	N/F	N/F	N/F	N/F	N/F*	14.15*(12.73)*	N/F*	N/F*
$\varepsilon_{D}^{ + }$	10.77*(8.35)*	N/F	N/F	N/F	N/F	N/F	N/F	N/F	N/F*
$\beta_{L}^{ - }$	8.50*(5.81)*	**8.24*(6.44)***	10.95*(9.36)*	N/F	14.21*(11.47)*	N/F	9.73*(8.89)*	N/F	8.31*(7.66)*
$\gamma_{L}^{ - }$	16.87*(14.27)*	11.98*(10.81)*	**14.38*(12.66)***	N/F	15.74*(14.76)*	N/F	13.33*(12.94)*	8.13*(8.04)*	N/F
$\gamma_{D}^{ - }$	N/F	**0.00*(0.00)***	2.30*(2.13)*	N/F	N/F	N/F	4.03*(3.88)*	N/F	N/F
$\delta_{L}^{ - }$	N/F	8.49*(6.67)*	11.09*(9.18)*	N/F*	14.51*(14.07)*	N/F	N/F	N/F	12.30*(11.28)*
$\delta_{D}^{ - }$	N/F	N/F	N/F	N/F*	N/F	N/F*	N/F	N/F*	N/F*
$\alpha_{L}^{ - }$	**9.18*(7.97)***	**8.79*(8.43)***	11.19*(10.61)*	N/F	N/F*	N/F	N/F	N/F*	N/F
$\alpha_{D}^{ - }$	N/F	N/F	N/F	N/F	N/F	N/F	**9.61*(8.52)***	4.41*(3.53)*	N/F*
$\varepsilon_{L}^{ - }$	N/F	N/F	N/F	N/F	N/F*	N/F*	N/F	N/F*	N/F*
$\varepsilon_{D}^{ - }$	N/F	N/F	N/F	N/F	N/F	N/F	N/F	N/F*	N/F

The bold values correspond to the relative energy and Gibbs free energies of *β*-turns in the gas phase, obtained at the B3LYP‒D3/6‒311 + G (*d,p*) level of theory

The calculated energies are relative to the minimum energy conformer, $\gamma_{D}^{ - } \gamma_{L}$. The values in parentheses are the relative Gibbs free energies. The N/F symbol indicates not found conformers migrated to different conformations. The starred N/F* show that both serine and alanine residues have migrated to different conformations. (Refer to Additional file 1: Table S1). BB, backbone; a, anti; + , gauche ( +); − , gauche ( −).

Based on the former study, the most stable and unstable BB conformers of l form of ser‒ala protected dipeptide were determined as the $\gamma_{L}^{ + } \gamma_{D}^{{}}$ and $\delta_{D}^{ - } \varepsilon_{L}$, respectively. Also, for the anti, gauche ( +), and gauche ( −), conformers of l‒ser‒l-ala protected dipeptide, ($\gamma_{L}^{a} \gamma_{D}$, $\alpha_{L}^{a} \gamma_{L}$), ($\gamma_{L}^{ + } \gamma_{D}$, $\gamma_{D}^{ + } \beta_{L}$), and ($\gamma_{L}^{ - } \gamma_{D}$, $\delta_{D}^{ - } \varepsilon_{L}$) BB conformers bear the highest and lowest stability, respectively [26]. As well as, l and d enantiomers of some similar protected dipeptides such as MeCO‒ala‒ala‒NHMe and l form of AC‒valyl‒alanine‒NHMe have presented $\beta_{L} \gamma_{L}$, $\beta_{L} \gamma_{D}$ and $\alpha_{D}^{a} \gamma_{L}$ conformers as the most stable species with the lowest potential energy minima in the gas phase, respectively [22, 60]. Moreover, for ala‒gly dipeptide it was found that the most stable conformations within gas phase and water solvent were adopted by $\gamma_{L}^{{}} \gamma_{D}^{{}}$ and $\varepsilon_{L} \delta_{D}^{{}}$ ones [60, 61].

### β − turn conformers

*Β* − turns have been classified and summarized based on the dihedral angle values ($\varphi$**,**$\psi$**)** of the (*i* + 1)th and (*i* + 2)th positions. It can result that, 22 cases of the 87 found conformations of For‒d‒ser‒ d‒ala‒NH_2_ can be categorized as the *β* − turns. All conformers of *β* − turns were optimized and verified to be at the minimum energy through vibrational analysis (no imaginary frequency mode). The optimizations of all 22 *β* − turns structures and frequency calculations were carried out at the B3LYP, B3LYP‒D3 and M06‒2X levels using the standard 6‒311 + G (*d,p*) basis set in gas and solution phases. To study the effect of solvent on the stability of *β* − turn structures, the quantum mechanical calculations were performed in water using Tomasi’s polarized continuum model at the M06‒2X/6–311 + G (*d,p*) level of theory.

Thermodynamic properties, including relative and Gibbs free energies, as well as the type of *β* − turns in gas and solution phases at the B3LYP‒D3/6–311 + G (*d,p*) and M06‒2X/6–311 + G (*d,p*) levels of theory, are provided in Table 4. Additionally, the classification of *β* − turns according to their type, torsional dihedrals ($\varphi$ and $\psi$), and their dipole moments (μ) in Debye, at the B3LYP‒D3 and M06‒2X/6–311 + G (*d,p*) levels of theory in the gas and water phases, was reported in the supplementary information (Additional file 1: Table S2).

**Table 4:** Calculated (B3LYP‒D3 and M06‒2X/6‒311 + G (*d,p*)) relative and Gibbs free energy values (kcalmol^−1^) of *β* − turn conformers of sa‒protected dipeptide in the gas and water phases

*β* − turn type	Conformer	B3LYP-D3		M06-2X (gas phase)		M06-2X (water)	
		**ΔE**	**ΔG**	**ΔE**	**ΔG**	**ΔE**	**ΔG**
I	$\alpha_{L}^{a} \gamma_{L}$	5.74	5.43	5.01	5.01	1.54	1.53
	$\alpha_{L}^{ + } \gamma_{L}$	7.45	6.57	6.65	6.14	3.56	3.42
	$\alpha_{L}^{ - } \gamma_{L}$	7.75	7.42	6.61	6.87	3.00	3.40
I'	$\alpha_{D}^{a} \gamma_{D}$	14.69	13.07	14.64	12.82	6.44	5.65
	$\alpha_{D}^{ + } \gamma_{D}$	9.22	8.01	9.39	8.33	4.92)	4.12
II	$\varepsilon_{L}^{a} \gamma_{D}$	8.87	8.20	8.89	7.77	5.37	4.75
	$\varepsilon_{L}^{a} \delta_{D}$	12.84	11.48	12.22	11.06	7.08	5.53
III'	$\alpha_{D}^{a} \alpha_{D}$	13.67	12.44	12.87	12.13	3.29	3.72
	$\alpha_{D}^{ + } \alpha_{D}$	7.37	7.07	6.36	6.31	0.98	0.96
	$\alpha_{D}^{ - } \alpha_{D}$	6.88	6.32	6.18	5.77	0.47	0.66
V	$\gamma_{L}^{a} \gamma_{D}$	10.03	9.19	10.87	9.86	7.08	6.58
	$\gamma_{L}^{ + } \gamma_{D}$	8.40	7.74	9.50	8.76	7.05	6.31
	$\gamma_{L}^{ - } \gamma_{D}$	12.62	11.60	13.80	12.23	9.60	8.57
V'	$\gamma_{D}^{a} \gamma_{L}$	2.91	2.48	3.01	2.81	1.73	1.74
	$\gamma_{D}^{ + } \gamma_{L}$	4.90	4.20	5.12	4.48	3.07	1.79
	$\gamma_{D}^{ - } \gamma_{L}$	0.00	0.00	0.00	0.00	0.00	0.00
VIa1	$\varepsilon_{L}^{a} \gamma_{L}$	7.18	6.43	6.79	6.01	3.54	3.09
VIa2	$\beta_{L}^{\alpha } \gamma_{L}$	3.46	2.46	3.26	2.08	2.00	1.28
	$\beta_{L}^{ - } \gamma_{L}$	6.69	5.12	6.37	5.08	2.99	2.25
VIII	$\alpha_{L}^{a} \beta_{L}$	6.30	5.14	5.59	4.52	1.52	0.49
	$\alpha_{L}^{ + } \beta_{L}$	8.56	6.84	7.93	6.66	3.75	2.49
	$\alpha_{L}^{ - } \beta_{L}$	8.43	7.27	7.25	6.53	2.95	2.52

The given data of Gibbs free energy in the gas phase indicate that, *β* − turn conformers that belong to types *V'* and *I'* have the highest and lowest stability, respectively. Nevertheless, based on the water solution calculations, it can be noted that $\gamma_{L}^{ - } \gamma_{D}^{{}}$ conformer as a type *V β* − turn is the highest unstable with the relative and Gibbs free energies of 9.60 and 8.57 kcalmol^−1^, respectively (see Table 4).

As shown in Tables 5 and 6, in the gas phase, the $\alpha_{D}^{a} \gamma_{D}$ conformer with the highest relative and Gibbs free energy should be considered as the lowest stable specie calculated at the B3LYP, B3LYP‒D3 and M06‒2X level of theory. The values of relative and Gibbs free energies of the $\alpha_{D}^{a} \gamma_{D}$ conformer in the gas phase at the B3LYP, B3LYP‒D3 and M06−2X levels of theory are calculated as 16.91 (14.85), 14.69 (13.07) and 14.64 (12.82) kcalmol^−1^, respectively. The values in parentheses are the relative Gibbs free energies. The thermodynamic properties of *β*−turns in the gas phase, obtained at the B3LYP/6‒311 + G (*d,p*) level of theory are shown in Table 3 (highlighted in bold).

However, the results of the solution phase showed that all *β*−turn structures have lesser relative energies and the higher stability. The energy decreasing by a dimensionless factor of 1/ε, (ε is the dielectric constant), is appropriate if the polarized or charged particles are immersed in any medium other than a vacuum. It can be mentioned that a weaker interaction in a polarizable medium (e.g., water) than a vacuum should be formed [62]. Polar molecules with a dipole moment (*μ)*, are dissolved in polar liquids. The dipole‒dipole interaction between solvent and solute can decrease the relative energies [63]. The order of stability of *β*−turn structures in the solvent and gas phase is different, which is due to the effect of solvent dielectric coefficient. For example, in the gas phase,$\alpha_{D}^{a} \gamma_{D}$ conformer is the most unstable structure, while in the solvent phase, $\gamma_{L}^{ - } \gamma_{D}^{{}}$ has the highest relative energy as the most unstable *β*−turn. Increasing the dipole moment of the $\alpha_{D}^{a} \gamma_{D}$ and $\gamma_{L}^{ - } \gamma_{D}^{{}}$ conformers to the values of 3.04 and 2.43 Debye, led to the decreasing down of relative and Gibbs free energies of 8.20 (7.17) and 4.20 (3.66) kcalmol^−1^, respectively. Therefore, the $\alpha_{D}^{a} \gamma_{D}$ conformer is more stable than $\gamma_{L}^{ - } \gamma_{D}^{{}}$ in water solvent (ref. Table 4).

The *β* − turn structures are stabilized by forming IHBs known as a $1 \leftarrow 4$ HB type, between the NH−function of the first amino acid ($ith$) residue and the carbonyl group of the fourth amino acid ($i + 3th$) residue. However, it was stipulated by Venkatachalam that 25% of *β*−turns are open, which means without any intraturn HB [41].

The theory of QTAIM provides a proper approach to elucidate the intermolecular interactions by considering the total electronic density,$\rho \left( {r_{c} } \right)$, and its corresponding Laplacian,$\nabla^{2} \rho \left( {r_{c} } \right)$.The AIM calculations based on Bader theory were performed for studying of HB interactions, including the topological analysis regarding the properties of bond critical points (BCPs) [64–67]. The $\rho \left( {r_{c} } \right)$,and $\nabla^{2} \rho \left( {r_{c} } \right)$ of BCPs describe the nature of HB and their values are related to the intermolecular interaction intensity. The greater *ρ* value, the more tremendous interaction energy and the positive $\nabla^{2} \rho \left( {r_{c} } \right)$ values indicate that the interaction is electrostatic (ionic interactions, HBs, and halogen bonds), while negative Laplacian values demonstrate the characteristic covalent interaction.

The high value of $\rho \left( {r_{c} } \right)$ at BCP of order > 10^–1^ a.u. along with the negative value of $\nabla^{2} \rho \left( {r_{c} } \right)$ indicating the presence of covalent interaction [68]. Nevertheless, the positive values of $\nabla^{2} \rho \left( {r_{c} } \right)$ along with the $\rho \left( {r_{c} } \right)$ amounts of 0.001 and 0.01 or less can be regarded for weak *van der Waals* and HB interactions, respectively [64, 67, 69]. The values of $\rho \left( {r_{c} } \right)$ and $\nabla^{2} \rho \left( {r_{c} } \right)$ at the BCP for the HB are in the range of 0.002–0.034 a.u. and 0.024–0.139 a.u., respectively [70].

The topological parameters and IHB energy (E_HB_) of *β*‒turn structures at the B3LYP‒D3 and M06‒2X levels of theory using the 6‒311 + G (*d,p*) basis set are summarized in Table 5. Only HB interactions were investigated, and unconventional interactions were ignored. Our results show that the E_HB_ of $O_{11} \cdots H_{19} - N_{9}$ (C10) (see Scheme 2), for $\alpha_{D}^{a} \alpha_{D}$,$\alpha_{D}^{ + } \alpha_{D}$ and $\alpha_{D}^{ - } \alpha_{D}$ conformers are measured as 3.62, 3.67, 2.62, and 3.72, 3.52, 2.78 kcal mol^−1^ with distance (r) of 2.07, 2.06, 2.18, and 2.07, 2.09, 2.17 Å at the B3LYP‒D3 and M06‒2X levels of theory, respectively that are in agreement with previous studies, (the NH···OC distance in C10 structures is around 2.10 − 2.20 Å) [71, 72]. These data show that increasing of $O_{11} \cdots H_{19}$ distance resulted in the diminishing of HB energy. The IHB energy is obtained by relation of $E_{HB} \, = \,\frac{1}{2}\,V\,(r_{BCP} )$ where $V\,(r_{BCP} )$ is the potential electronic energy density at the critical point [67]. In the investigated *β*‒turn structures, different types of HBs including $O_{14} \cdots H_{19} - N_{9}$(C7_A_), $O_{11} \cdots H_{15} - N_{6}$(C7_F_), $O_{14} \cdots H_{12} - N_{3}$(C5_S_), $O_{18} \cdots H_{15} - N_{6}$(C5_A_), $O_{14} \cdots H_{27} - O_{21}$(${}^{\gamma }6$), $O_{21} \cdots H_{15} - N_{6}$($6^{\gamma }$), and $N_{3} \cdots H_{15} - N_{6}$($5^{N}$), were observed (see Fig. 1 and Scheme 2).

**Table 5:** Calculated (B3LYP‒D3/6‒311 + G (*d,p*) and M0‒2X/6‒311 + G (*d,p*)) topological parameters (in a.u.) and IHB energy (in kcal mol^−1^) for *β*-turn conformers of sa-protected dipeptide

β-turn type	Conformer	Method	$O_{14} \cdots H_{19} - N_{9}$				$N_{3} \cdots H_{15} - N_{6}$				$O_{14} \cdots H_{27} - O_{21}$			
	Topological parameters		$r$	$\rho (r)$	$\nabla^{2} \rho (r)$	$E_{HB}$	$r$	$\rho (r)$	$\nabla^{2} \rho (r)$	$E_{HB}$	$r$	$\rho (r)$	$\nabla^{2} \rho (r)$	$E_{HB}$
I	$\alpha_{L}^{a} \gamma_{L}$	B3LYP	2.22	0.015	0.051	2.95	–	–	–	–	2.10	0.021	0.072	4.83
		M06-2X	2.18	0.016	0.058	3.38	–	–	–	–	2.08	0.021	0.080	5.34
	$\alpha_{L}^{ + } \gamma_{L}$	B3LYP	2.20	0.015	0.053	3.07	–	–	–	–	–	–	–	–
		M06-2X	2.17	0.016	0.058	3.35	–	–	–	–	–	–	–	–
	$\alpha_{L}^{ - } \gamma_{L}$	B3LYP	2.25	0.014	0.047	2.74	–	–	–	–	2.42	0.011	0.040	2.53
		M06-2X	2.20	0.015	0.054	3.17	–	–	–	–	2.33	0.013	0.048	3.07
I'	$\alpha_{D}^{a} \gamma_{D}$	B3LYP	1.99	0.023	0.085	5.22	–	–	–	–	–	–	–	–
		M06-2X	2.04	0.020	0.079	4.59	–	–	–	–	–	–	–	–
	$\alpha_{D}^{ + } \gamma_{D}$	B3LYP	1.98	0.024	0.087	5.42	2.29	0.017	0.070	3.72	–	–	–	–
		M06-2X	2.00	0.022	0.089	5.31	2.28	0.018	0.076	4.10	–	–	–	–
			$O_{14} \cdots H_{19} - N_{9}$				$O_{21} \cdots H_{15} - N_{6}$							
II	$\varepsilon_{L}^{a} \gamma_{D}$	B3LYP	1.98	0.024	0.088	5.51	2.00	0.024	0.091	5.70	–	–	–	–
		M06-2X	2.02	0.022	0.085	5.03	2.01	0.023	0.092	5.60	–	–	–	–
	$\varepsilon_{L}^{a} \delta_{D}$	B3LYP	–	–	–	–	2.02	0.023	0.088	5.39	–	–	–	–
		M06-2X	–	–	–	–	2.03	0.022	0.090	5.40	–	–	–	–
			$$O_{11} \cdots H_{19} - N_{9}$$											
III'	$\alpha_{D}^{a} \alpha_{D}$	B3LYP	2.07	0.017	0.068	3.62	–	–	–	–	–	–	–	–
		M06-2X	2.07	0.017	0.071	3.72	–	–	–	–	–	–	–	–
	$\alpha_{D}^{ + } \alpha_{D}$	B3LYP	2.06	0.018	0.069	3.67	–	–	–	–	–	–	–	–
		M06-2X	2.09	0.016	0.067	3.52	–	–	–	–	–	–	–	–
	$\alpha_{D}^{ - } \alpha_{D}$	B3LYP	2.18	0.014	0.050	2.62	–	–	–	–	–	–	–	–
		M06-2X	2.17	0.014	0.054	2.78	–	–	–	–	–	–	–	–
			$O_{14} \cdots H_{19} - N_{9}$				$O_{11} \cdots H_{15} - N_{6}$							
V	$\gamma_{L}^{a} \gamma_{D}$	B3LYP	1.94	0.026	0.095	6.16	2.00	0.023	0.083	5.20	–	–	–	–
		M06-2X	2.07	0.024	0.097	6.03	2.06	0.021	0.083	5.01	–	–	–	–
	$\gamma_{L}^{ + } \gamma_{D}$	B3LYP	1.95	0.026	0.094	5.96	1.89	0.030	0.103	7.16	–	–	–	–
		M06-2X	1.96	0.024	0.097	6.00	1.93	0.026	0.101	6.52	–	–	–	–
	$\gamma_{L}^{ - } \gamma_{D}$	B3LYP	1.94	0.027	0.097	6.32	1.81	0.036	0.121	9.35	–	–	–	–
		M06-2X	1.95	0.025	0.100	6.25	1.84	0.032	0.121	8.56	–	–	–	–
			$O_{14} \cdots H_{19} - N_{9}$				$O_{14} \cdots H_{27} - O_{21}$				$O_{11} \cdots H_{15} - N_{6}$			
V'	$\gamma_{D}^{a} \gamma_{L}$	B3LYP	2.09	0.019	0.070	4.18	2.19	0.018	0.061	4.02	2.05	0.021	0.075	4.50
		M06-2X	2.07	0.020	0.077	4.56	2.13	0.019	0.073	4.78	2.06	0.020	0.077	4.51
			$O_{14} \cdots H_{19} - N_{9}$				$O_{14} \cdots H_{27} - O_{21}$				$O_{11} \cdots H_{15} - N_{6}$			
	$\gamma_{D}^{ - } \gamma_{L}$	B3LYP	2.10	0.019	0.068	4.05	2.15	0.023	0.079	4.33	2.06	0.022	0.076	4.34
		M06-2X	2.08	0.019	0.075	4.41	2.12	0.020	0.073	4.84	2.06	0.020	0.076	4.53
			$O_{14} \cdots H_{19} - N_{9}$				$O_{21} \cdots H_{15} - N_{6}$				$O_{11} \cdots H_{15} - N_{6}$			
	$\gamma_{D}^{ + } \gamma_{L}$	B3LYP	2.07	0.020	0.072	4.34	–	–	–	–	1.96	0.025	0.091	5.81
		M06-2X	2.06	0.020	0.078	4.63	–	–	–	–	1.97	0.024	0.095	5.91
VIa1	$\varepsilon_{L}^{a} \gamma_{L}$	B3LYP	2.08	0.020	0.071	4.26	2.02	0.023	0.087	5.34	–	–	–	–
		M06-2X	2.06	0.020	0.077	4.55	2.03	0.022	0.089	5.32	–	–	–	–
			$O_{14} \cdots H_{19} - N_{9}$				$O_{14} \cdots H_{12} - N_{3}$				$O_{21} \cdots H_{15} - N_{6}$			
VIa2	${\beta }_{L}^{\alpha }{\gamma }_{L}$	B3LYP	2.10	0.019	0.068	4.01	2.14	0.021	0.095	5.12	2.02	0.022	0.088	5.19
		M06-2X	2.07	0.019	0.075	4.44	2.13	0.022	0.104	5.61	2.02	0.022	0.093	5.36
	${\beta }_{L}^{-}{\gamma }_{L}$	B3LYP	2.12	0.018	0.065	3.84	–	–	–	–	–	–	–	–
		M06-2X	2.09	0.019	0.073	4.27	–	–	–	–	–	–	–	–
			$O_{18} \cdots H_{15} - N_{6}$				$O_{14} \cdots H_{27} - O_{21}$							
VIII	$\alpha_{L}^{a} \beta_{L}$	B3LYP	–	–	–	–	2.06	0.022	0.077	5.24	–	–	–	–
		M06-2X	–	–	–	–	2.05	0.022	0.084	5.61	–	–	–	–
	$\alpha_{L}^{ - } \beta_{L}$	B3LYP	2.15	0.020	0.095	4.98	2.38	0.012	0.042	2.68	–	–	–	–
		M06-2X	2.12	0.021	0.105	5.56	2.31	0.013	0.050	3.16	–	–	–	–
	$\alpha_{L}^{ + } \beta_{L}$	(No intramolecular interactions including hydrogen bonding or other types were observed)												

According to Rozas et al. HBs can be classified into weak, medium, and strong based on $\rho \left( {r_{c} } \right)$,$\nabla^{2} \rho \left( {r_{c} } \right)$, and E_HB_ values [70]. The measured values of $\rho_{O \cdots H}$ for $O_{14} \cdots H_{19} - N_{9}$ in different *β*‒turn conformers in the ranges of 0.014‒0.027 and 0.015‒0.025 a.u. and $\nabla^{2} \rho_{O \cdots H}$ in the contents of 0.047‒0.097 and 0.054‒0.100 a.u. made E_HB_ amounts in the range of 2.74‒6.32 and 3.17‒6.25 kcal mol^−1^ at the B3LYP‒D3 and M06‒2X levels of theory, respectively. The highest and lowest amounts of $\rho \left( {r_{c} } \right)$,$\nabla^{2} \rho \left( {r_{c} } \right)$, and E_HB_ for C7_A_ IHB belonging to the $\gamma_{L}^{ - } \gamma_{D}$ and $\alpha_{L}^{ - } \gamma_{L}$ of types V and I *β*‒turn secondary structures, respectively. As shown in Table 5 the B3LYP‒D3 and M06‒2X values of $\rho \left( {r_{c} } \right)$,$\nabla^{2} \rho \left( {r_{c} } \right)$, and E_HB_ are in the ranges of (0.011‒0.036 a.u.), (0.040‒0.121 a.u.), (2.53‒9.35 kcal mol^−1^) and (0.013‒0.032 a.u.), (0.048‒0.121 a.u.), (2.78‒8.56 kcal mol^−1^), respectively. The residues possessing an HB acceptor and/or donor site potentially lead to a NH → side chain and/or side chain donor → backbone CO HB that are observed concomitantly with backbone − backbone HBs. This is the case for instance with the Ser, His, Asn, and Gln side chains, where an extended locking through multiple bonds occurs between the backbone and the side chain [40]. For ser‒ala protected dipeptide the side chain of ser residue could create IHBs such as Cn (n = 5, 7 and 10),${}^{\gamma }6$ and $6^{\gamma }$. The obtained results at the B3LYP‒D3 and M06‒2X levels of theory show that the ${}^{\gamma }6$ and C10 IHBs of $\alpha_{L}^{ - } \gamma_{L}$ and $\alpha_{D}^{ - } \alpha_{D}$ *β*‒turns have lowest E_HB_, whereas, C7_F_ interaction of $\gamma_{L}^{ - } \gamma_{D}$ *β*‒turn, show the highest one, respectively (see Table 5). The $\gamma_{D}^{ - } \gamma_{L}$ conformer of type V' *β*‒turn is the most stable one, including three IHBs: a one ${}^{\gamma }6$ and two C7 interactions whatever computed at the B3LYP‒D3 and M06‒2X levels of theory. These IHBs (C7_A_, C7_F_, and ${}^{\gamma }6$) have E_HB_ values and $O \cdots H$ distances obtained as (4.05, 4.33, and 4.34 kcal mol^−1^), (2.10, 2.06, and 2.15 Å) and (4.41, 4.53, and 4.84 kcal mol^−1^), (2.08, 2.06, and 2.12 Å) at the B3LYP‒D3 and M06‒2X levels of theory, respectively (Tables 4 and 5).

It should be noted that although one of the γ‒turn characteristics is the creation of C7 IHB, some studies show that this bond and also ${}^{\gamma }6$ and $5^{\gamma }$ interactions are observed in both γ‒ and *β*‒turns. [40, 46, 49]. However, our results show that the most unstable $\alpha_{D}^{a} \gamma_{D}$ *β*-turn structure of type I' has one C7_A_ with calculated E_HB_ values as 5.22 and 4.59 kcal mol^−1^ and r distance in the amount of 1.99 and 2.04 Å at B3LYP-D3 and M06-2X levels of theory, respectively. The AIM calculations show that there are not any interactions for $\alpha_{L}^{ + } \beta_{L}$ *β*‒turn structure.

**Fig. 4:**
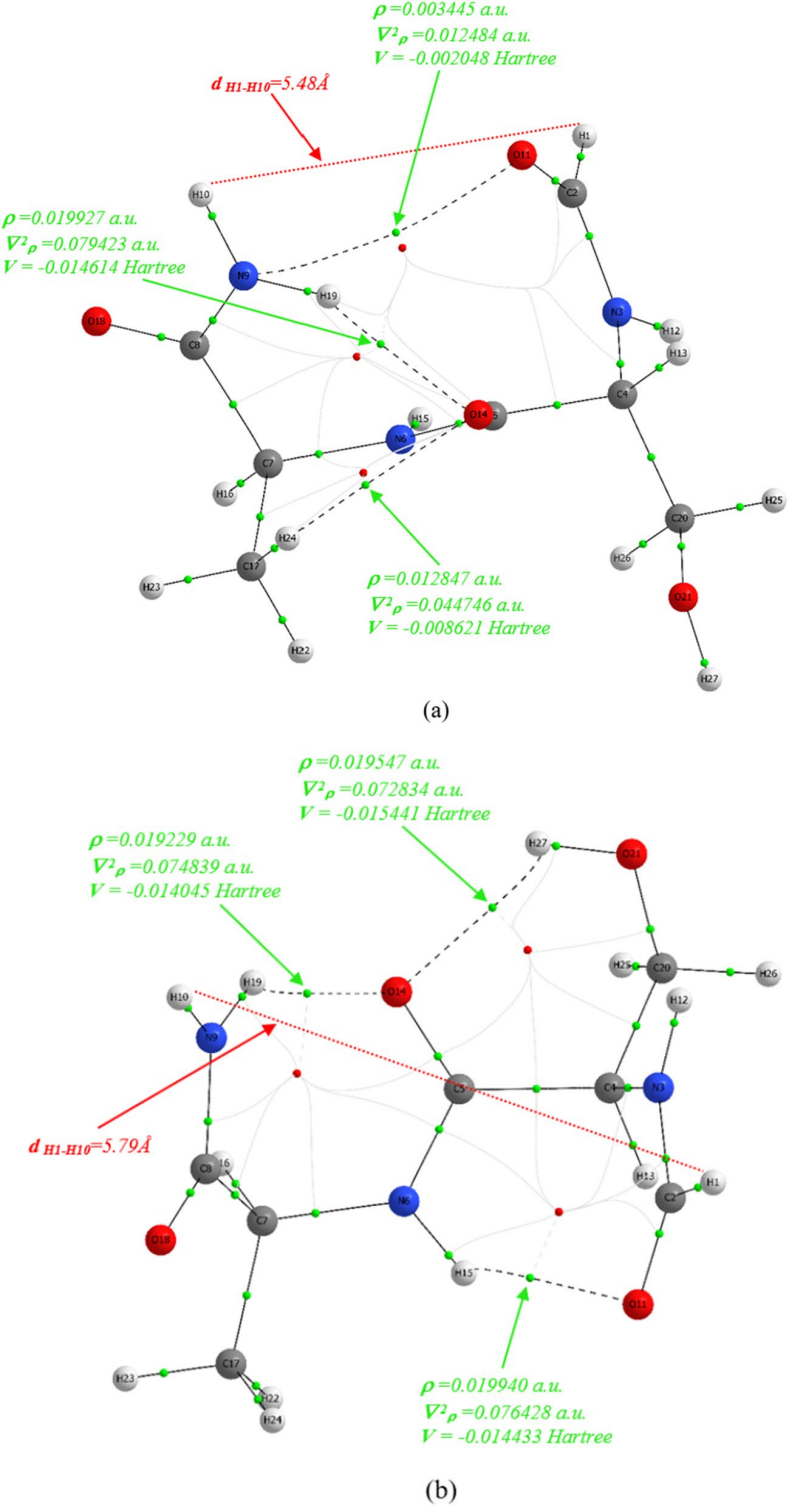
The electron density, Laplacian, and potential electronic energy values (in a.u.) for two* β*‒turn conformers of the HCO‒D‒ser‒D‒ala‒NH_2_ protected dipeptide at the M06‒2X/6‒311 + G (*d,p*) level of theory. **a** $\alpha_{D}^{a} \gamma_{D}$ **b** $\gamma_{D}^{ - } \gamma_{L}$. The small green dots represent the BCPs

The most stable and unstable *β*‒turn structures,$\gamma_{D}^{ - } \gamma_{L}$ and $\alpha_{D}^{a} \gamma_{D}$, of the sa‒protected dipeptide are shown in Fig. 4. For* β*‒turn conformers the distance between H_1_ and H_10_ atoms, ($d_{{H_{1} - H_{10} }}$), of HCO and NH_2_ protecting groups must be < 7 Å except for VIa1, VIa2, and VIb types that $d_{{H_{1} - H_{10} }}$ would be greater than 7 Å [73]. The value of $d_{{H_{1} - H_{10} }}$ for $\gamma_{D}^{ - } \gamma_{L}$ and $\alpha_{D}^{a} \gamma_{D}$ conformers was measured as 5.75, 5.93 Å and 5.79, 5.48 Å at the B3LYP‒D3 and M06‒2X levels of theory, respectively (see Fig. 4). The range of $d_{{H_{1} - H_{10} }}$ is from 4.94 ($\alpha_{L}^{ - } \gamma_{L}$- type I) to 8.71 Å ($\varepsilon_{L}^{a} \delta_{D}$-type II) and 4.83 ($\alpha_{L}^{ - } \gamma_{L}$- type I) to 8.78 Å (${\beta }_{L}^{-}{\delta }_{L}$-type VIa2) for the gas phase computed conformers at the B3LYP‒D3 and M06‒2X levels of theory, respectively.

It could be pointed out that the calculations of solution media show that the distance between H_1_ and H_10_ atoms is in the range of 5.25‒8.73 Å for the $\alpha_{D}^{a} \alpha_{D}$(type III') and $\varepsilon_{L}^{a} \delta_{L}$(type VIa1) *β*‒turns, respectively. The values of the electron density, Laplacian, and potential electronic energy, V(r), (in a.u.) at the M06‒2X level of theory for three conventional C7_A_, C7_F_,${}^{\gamma }6$ and two unconventional ($N_{9} \cdots O_{11} - C_{2}$) and ($O_{14} \cdots H_{24} - C_{17}$) IHBs for the $\gamma_{D}^{ - } \gamma_{L}$ and $\alpha_{D}^{a} \gamma_{D}$* β*-turns are shown in Fig. 4. As shown in Fig. 4a the values of V(r) for C7_A_, $O_{14} \cdots H_{24} - C_{17}$, and $N_{9} \cdots O_{11} - C_{2}$ IHB interactions at BCPs for the $\alpha_{D}^{a} \gamma_{D}$ conformer, are obtained as (-0.014614, -0.008621, -0.002048) Hartree, respectively. Therefore, the obtained E_HB_ for these interactions is (4.59, 2.70, 0.64) kcal mol^−1^, respectively. Also, the values of V(r) and E_HB_ for C7_A_, C7_F_,${}^{\gamma }6$ IHB interactions in $\gamma_{D}^{ - } \gamma_{L}$ conformer are calculated as (-0.014045, -0.014433, -0.015441, Hartree), (4.41, 4.53, 4.84 kcal mol^−1^), respectively (see Fig. 4b).

Our results show that the most unstable conformer of $\delta_{L}^{ + } \varepsilon_{D}$,is not in a *β*‒turn region of the Ramachandran map. While the $\gamma_{D}^{ - } \gamma_{L}$, *β*-turn conformer of type V' is the most stable conformer among all 87 considered ones with the three IHBs of C7_A_, C7_F_, and ${}^{\gamma }6$ (Table 5 and Fig. 4a). Therefore, the HCO − d − ser − d − ala − NH_2_ dipeptide adopt a *β*‒turn conformation.

### Study of electronic transitions: FMO analysis

Herein, the electronic study of the most stable conformer of HCO − d − ser − d − ala − NH_2_ protected dipeptide, $\gamma_{D}^{ - } \gamma_{L}$, has been considered using TD‒DFT calculations at the B3LYP‒D3/6‒311 + G (*d,p*) level of theory. The computed electronic transitions in vacuo by TD − DFT method show several sharp bands assigned to intra‒charge transfer (CT) in the respected dipeptide.

**Fig. 5:**
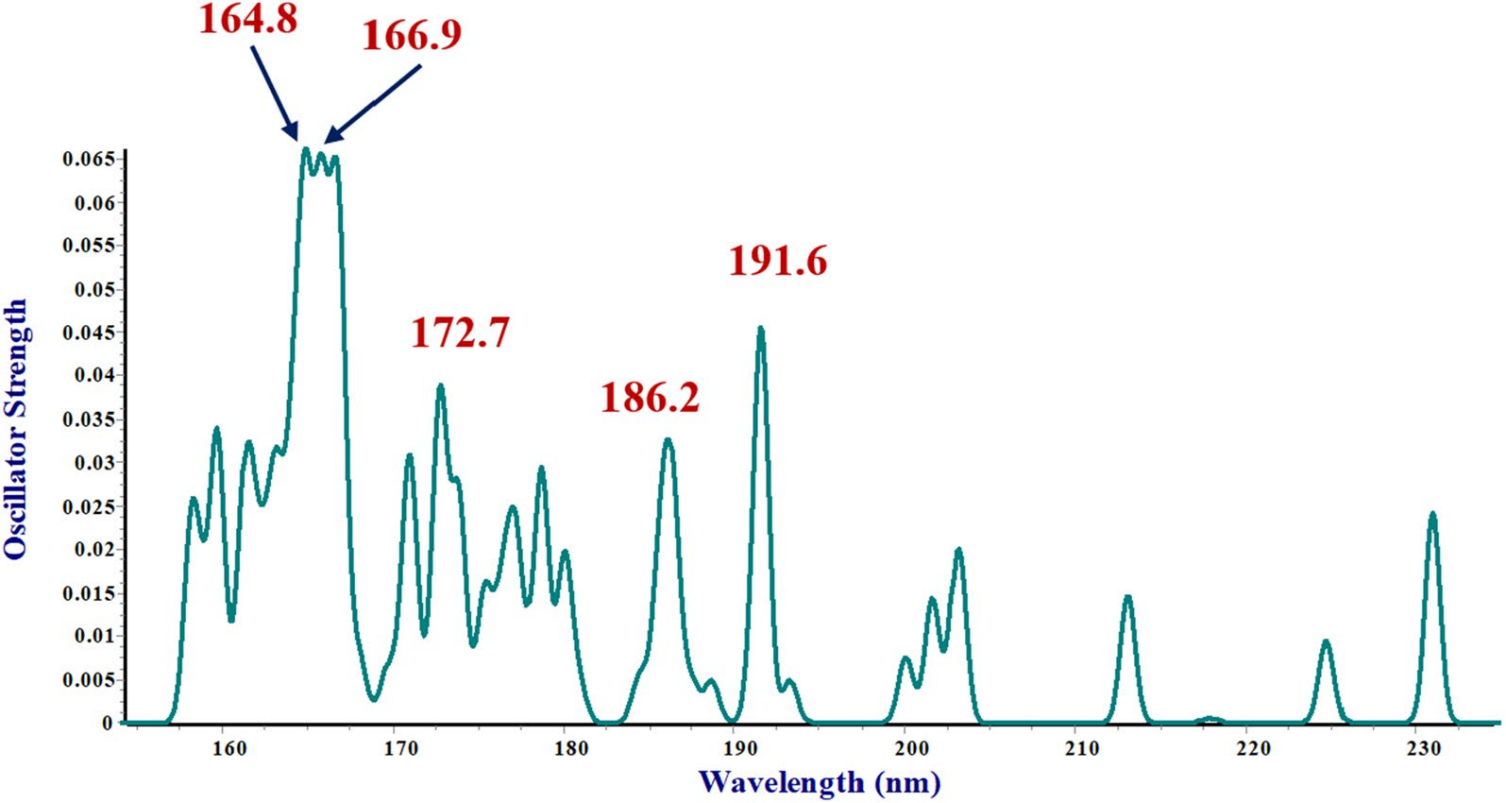
Calculated (B3LYP‒D3/6‒311 + G (*d,p*) electronic spectra for $\gamma_{D}^{ - } \gamma_{L}$ conformer of sa-protected dipeptide

**Fig. 6:**
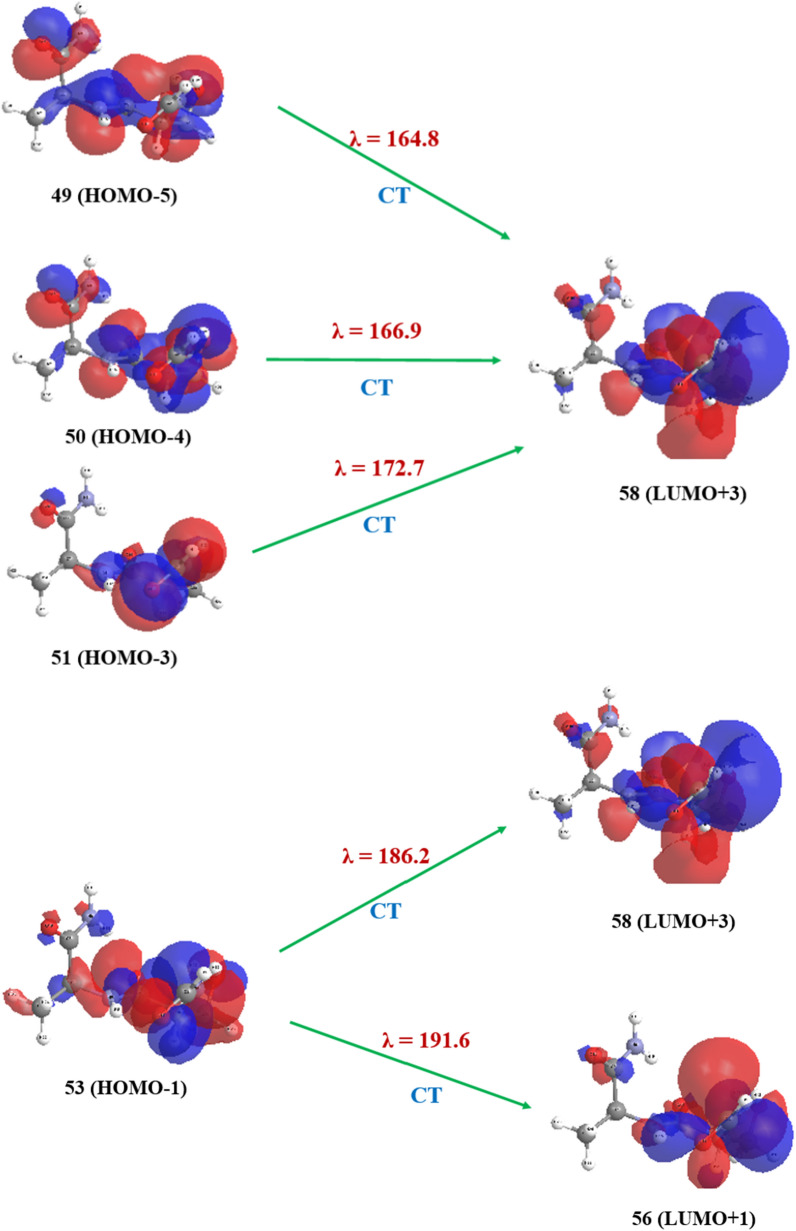
Frontier molecular diagrams for $\gamma_{D}^{ - } \gamma_{L}$ conformer of sa‒protected dipeptide involving in CT obtained according to the B3LYP‒D3/6‒311 + G (*d*, *p*) level of theory

However, the computed UV−Vis spectrum of sa‒protected dipeptide indicates several main CTs that five major ones can mention as 164.8, 166.9, 172.7, 186.2 and 191.6 nm (see Fig. 5). The electronic characteristic issue of optimized ground state structure of sa‒protected dipeptide has been performed by molecular orbital theoretical considerations along with natural population analysis (NPA). The optimized geometry bears singlet multiplicity due to 54 occupied molecular orbitals (MOs). The FMOs investigation was applied to elucidation and attributing that CTs to the pertinent UV − Vis calculated spectrum (see Table 6). Moreover, the accurately calculated and reported results of FMOs energy show the consistency and confirmation with herein obtained results [74, 75]. They used these data to find out the interaction sites of molecule with the other species. The lowest unoccupied molecular orbitals (LUMOs) and in some orbitals of highest occupied molecular orbitals (HOMOs) that involve in ET are populated over alanine residue of computed dipeptide. They are almost composed of just *s* and *p* orbitals. The 49 (HOMO−5), 50 (HOMO−4), 51 (HOMO‒3), and 53 (HOMO‒1) consist primarily of lone pair(s) electrons of atomic orbitals of oxygen atoms of alanine part of sa‒protected dipeptide (comprising ~ *s* 59.34%* p* 40.66; *p* ~ 100%; *s* 58.46% *p* 41.51%; and *s* 48.35%, *d* 51.65%, respectively) and 56 (LUMO + 1) and 58 (LUMO + 3) consist primarily of anti−bonding Rydberg molecular orbitals of hydrogen atoms of alanine moiety of sa‒protected dipeptide (comprising *s* 87.26% *p* 12.74%*;* and *s* 7.76%, *p* 92.24%, respectively). Therefore, it can be said that all of the computed ET bands can be assigned to the n → n^*^ intra−ligand charge transfer (ILCT) transitions (see Fig. 6). All the four bands (λ_1_, λ_2,_ λ_3,_ and λ_4_) at 164.8, 166.9, 172.7, and 186.2 are attributed to CT of intra transfer of alanine amino acid residue within CT from 49 (HOMO−5), 50 (HOMO−4), 51 (HOMO−3), and 53 (HOMO−1) to 58 (LUMO + 3), respectively. Also, the last one (λ5) at 191.6 nm could be assigned to CT of intra charge transition of alanine amino acid part of dipeptide from 53 (HOMO−1) to 56 (LUMO + 1).

**Table 6:** Calculated (B3LYP‒D3/6‒311 + G (*d,p*)) electronic data for $\gamma_{D}^{ - } \gamma_{L}$ conformer of sa‒protected dipeptide

Wave Length(nm)	ΔE_ad_ (ev)	Oscillator strength	Electronic transition	Involved orbitals in CTs	Calculated energies
164.8	7.52	0.049	49 (HOMO-5) → 58 (LUMO + 3)	HOMO (a.u.)	− 0.2589
166.9	7.43	0.039	50 (HOMO-4) → 58 (LUMO + 3)	LUMO (a.u.)	− 0.0276
172.7	7.18	0.036	51 (HOMO-3) → 58 (LUMO + 3)	Δ (LUMO–HOMO) (a.u.)	0.2313
186.2	6.66	0.021	53 (HOMO-1) → 58 (LUMO + 3)	HOMO-5 (a.u.)	− 0.3087
191.6	6.47	0.042	53 (HOMO-1) → 56 (LUMO + 1)	HOMO-4 (a.u.)	− 0.3031
				HOMO-3 (a.u.)	‒0.2951
				HOMO-1 (a.u.)	‒0.2774
				LUMO + 1 (a.u.)	− 0.0126
				LUMO + 3 (a.u.)	− 0.0034

## Conclusion

A complete conformational analysis of the d−ser−d−ala protected dipeptide was performed using B3LYP/6‒311 + G (*d,p*) calculations in the gas phase. In addition, all *β*−turn structures were optimized in the gas and solution phases and verified to be at the minimum energy using DFT‒B3LYP‒D3 and M06‒2X levels of theory. Also, the *β*−turn conformers have been considered at that those same levels of theory by using QTAIM. According to MDCA, the whole 3^5^ = 243 plausible conformers were investigated. The results show that 87 stable conformers were found and 156 ones changed to some different more stable conformations among the found conformations. It can be highlighted that $\gamma_{D}^{ - } \gamma_{L}$ and $\delta_{L}^{ + } \varepsilon_{D}$ were characterized as the most stable and unstable conformers, respectively. Also, based on the migration pattern of sa‒protected dipeptide, it can be suggested that ($\beta_{L}$,$\gamma_{D}$,$\gamma_{L}$) and ($\gamma_{L}$,$\gamma_{D}$) conformers of ser and ala residues provide the more stable dipeptide model. Moreover, 22 conformers of the all 87 found ones of For‒d‒ser‒d‒ala‒NH_2_ can be categorized as the *β*−turn structures bearing $\gamma_{D}^{ - } \gamma_{L}$ as the most stable conformer in the gas and water solvent phases.

The AIM calculations discover the formation of a C10 HB ($O_{11} \cdots H_{19} - N_{9}$) between the N‒H and C=O of residues *i* and *i* + 3 in $\alpha_{D}^{a} \alpha_{D}$,$\alpha_{D}^{ + } \alpha_{D}$ and $\alpha_{D}^{ - } \alpha_{D}$ *β*−turns with E_HB_ of 3.62, 3.67, 2.62, and 3.72, 3.52, 2.78 kcal mol^−1^ at the B3LYP‒D3 and M06‒2X levels of theory, respectively. The $\gamma_{D}^{ - } \gamma_{L}$ conformer of type V' *β*‒turn, is the most stable one that includes three IHBs (C7 and ${}^{\gamma }6$ types) with the calculated E_HB_ of (4.05, 4.33, & 4.34 kcal mol^−1^ and 4.41, 4.84, & 4.53 kcal mol^−1^at B3LYP‒D3 or M06‒2X levels of theory, respectively. However, the $\alpha_{D}^{a} \gamma_{D}$ conformer of type I'* β*‒turn, is the most unstable species in the gas phase and consists of one C7 HB with E_HB_ measured at the B3LYP and M06−2X levels of theory as 5.22 and 4.59 kcal mol^−1^, respectively. Finally, since $\gamma_{D}^{ - } \gamma_{L}$ as the most stable conformer of sa‒protected dipeptide is in a *β*‒turn region of the Ramachandran map, the HCO−d−ser−d−ala−NH_2_ dipeptide adopts a *β*‒turn conformation.

The calculated electronic spectrum by the TD‒DFT method for the most stable conformer of sa‒protected dipeptide indicated the five major bands. NBO and electronic transitions analysis of $\gamma_{D}^{ - } \gamma_{L}$ conformer show that the bands in the UV‒Vis spectrum are mainly attributed to n → n^*^ of intra‒transfer of alanine moiety of sa‒protected dipeptide.

### Supplementary Information

**Additional file 1: Table S1.** Initial and final conformations of For‒d‒ser‒d‒ala‒NH_2_ dipeptide for anti, gauche (+) and gauche (−) side-chain conformations. **Table S2.** Classification of *β*−turn conformers of sa-protected dipeptide according to their type, torsional dihedrals $\varphi_{i + 1}$, $\psi_{i + 1}$, $\varphi_{i + 2}$, $\psi_{i + 2}$ and dipole moment (*μ)* (Debye), at the B3LYP‒D3 and M06‒2X/6‒311+G (*d,p*) levels of theory in the gas and water phases.

## Data Availability

The authors certify that all used materials and reported data, as well as a software applications or custom codes support the published claims and comply with field standards.

## Abbreviations

ser: Serine
ala: Alanine
CNS: Central nervous system
sa: Ser‒ala
BB: Backbone
FMO: Frontier molecular orbital
PCM: Polarized continuum model
ZPE: Zero‒point vibrational energy
MDCA: Multidimensional conformational analysis
NBO: Natural bond orbital
QTAIM: Quantum theory of atoms in molecules
IHB: Intramolecular hydrogen bonds
PEHS: Potential energy hypersurface
CT: Charge transfer
NPA: Natural population analysis
MO: Molecular orbitals
HOMO: Highest occupied molecular orbital
LUMO: Lowest unoccupied molecular orbital
ILCT: Intra−ligand charge transfer
