# Supplementary material for: An accurate DFT study within conformational survey of the d-form serine−alanine protected dipeptide
Source: BMC Chem. 2023 Oct 13;17(1):138. doi: 10.1186/s13065-023-01051-9 (PMC10571400; doi:10.1186/s13065-023-01051-9)
Supplement: Supplementary file 1 — Additional file 1: Table S1. Initial and final conformations of For‒d‒ser‒d‒ala‒NH2 dipeptide for anti, gauche (+) and gauche (−) side-chain conformations. Table S2. Classification of β−turn conformers of sa-protected dipeptide according to their type, torsional dihedrals $\varphi_{i + 1}$φi+1, $\psi_{i + 1}$ψi+1, $\varphi_{i + 2}$φi+2, $\psi_{i + 2}$ψi+2 and dipole moment (μ) (Debye), at the B3LYP‒D3 and M06‒2X/6‒311+G (d,p) levels of theory in the gas and water phases. [file 13065_2023_1051_MOESM1_ESM.docx]

**Additional file**

**An Accurate DFT study within conformational survey of the D-form serine−alanine protected dipeptide**

**Behzad Chahkandi^[[1]](#footnote-1)a^ *, Mohammad Chahkandi ^b^**

^a^ Department of Chemistry, Mashhad Branch, Islamic Azad University, Mashhad, Iran

^b^ Department of Chemistry, Hakim Sabzevari University, Sabzevar 96179-76487, Iran

**Table S1.** Initial and final conformations of For‒D‒ser‒D‒ala‒NH_2_ dipeptide for anti, gauche (+) and gauche (−) side-chain conformations.

| ***anti (a)*** | | | | | | | | |
| --- | --- | --- | --- | --- | --- | --- | --- | --- |
| $\gamma_{D}\gamma_{D}$($\gamma_{D}\gamma_{D}$) | $\gamma_{D}\delta_{D}$($\gamma_{D}\delta_{D}$) | $\gamma_{D}\alpha_{L}$($\gamma_{D}\delta_{D}$) | $\delta_{D}\gamma_{D}$($\beta_{L}\gamma_{D}$) | $\delta_{D}\delta_{D}$($\beta_{L}\delta_{D}$) | $\delta_{D}\alpha_{L}$($\beta_{L}\delta_{L}^{*}$) | $\alpha_{L}\gamma_{D}$($\alpha_{L}\gamma_{D}$) | $\alpha_{L}\delta_{D}$($\alpha_{L}\delta_{D}$) | $\alpha_{L}\alpha_{L}$($\alpha_{L}\gamma_{L}$) |
| $\gamma_{D}\varepsilon_{D}$($\gamma_{D}\gamma_{D}$) | $\gamma_{D}\beta_{L}$($\gamma_{D}\beta_{L}$) | $\gamma_{D}\varepsilon_{L}$($\gamma_{D}\gamma_{L}$) | $\delta_{D}\varepsilon_{D}$($\beta_{L}\varepsilon_{D}$) | $\delta_{D}\beta_{L}$($\beta_{L}\beta_{L}$) | $\delta_{D}\varepsilon_{L}$($\delta_{D}\varepsilon_{L}$) | $\alpha_{L}\varepsilon_{D}$($\alpha_{L}\gamma_{D}$) | $\alpha_{L}\beta_{L}$($\alpha_{L}\beta_{L}$) | $\alpha_{L}\varepsilon_{L}$($\alpha_{L}\beta_{L}$) |
| $\gamma_{D}\alpha_{D}$($\gamma_{D}\alpha_{D}$) | $\gamma_{D}\delta_{L}$($\gamma_{D}\delta_{D}$) | $\gamma_{D}\gamma_{L}$($\gamma_{D}\gamma_{L}$) | $\delta_{D}\alpha_{D}$($\beta_{L}\alpha_{D}$) | $\delta_{D}\delta_{L}$($\beta_{L}\delta_{L}$) | $\delta_{D}\gamma_{L}$($\beta_{L}\gamma_{L}$) | $\alpha_{L}\alpha_{D}$($\alpha_{L}\gamma_{D}$) | $\alpha_{L}\delta_{L}$($\alpha_{L}\gamma_{L}$) | $\alpha_{L}\gamma_{L}$($\alpha_{L}\gamma_{L}$) |
| $\varepsilon_{D}\gamma_{D}$($\beta_{L}\gamma_{D}$) | $\varepsilon_{D}\delta_{D}$($\beta_{L}\delta_{D}$) | $\varepsilon_{D}\alpha_{L}$($\gamma_{D}\gamma_{D}^{*}$) | $\beta_{L}\gamma_{D}$($\beta_{L}\gamma_{D}$) | $\beta_{L}\delta_{D}$($\beta_{L}\delta_{D}$) | $\beta_{L}\alpha_{L}$($\beta_{L}\delta_{L}$) | $\varepsilon_{L}\gamma_{D}$($\varepsilon_{L}\gamma_{D}$) | $\varepsilon_{L}\delta_{D}$($\varepsilon_{L}\delta_{D}$) | $\varepsilon_{L}\alpha_{L}$($\varepsilon_{L}\delta_{L}$) |
| $\varepsilon_{D}\varepsilon_{D}$($\beta_{L}\varepsilon_{D}$) | $\varepsilon_{D}\beta_{L}$($\beta_{L}\beta_{L}$) | $\varepsilon_{D}\varepsilon_{L}$($\beta_{L}\gamma_{L}^{*}$) | $\beta_{L}\varepsilon_{D}$($\beta_{L}\varepsilon_{D}$) | $\beta_{L}\beta_{L}$($\beta_{L}\beta_{L}$) | $\beta_{L}\varepsilon_{L}$($\beta_{L}\gamma_{L}$) | $\varepsilon_{L}\varepsilon_{D}$($\varepsilon_{L}\varepsilon_{D}$) | $\varepsilon_{L}\beta_{L}$($\varepsilon_{L}\beta_{L}$) | $\varepsilon_{L}\varepsilon_{L}$($\varepsilon_{L}\gamma_{L}$) |
| $\varepsilon_{D}\alpha_{D}$($\beta_{L}\alpha_{D}$) | $\varepsilon_{D}\delta_{L}$($\beta_{L}\delta_{L}$) | $\varepsilon_{D}\gamma_{L}$($\beta_{L}\gamma_{L}$) | $\beta_{L}\alpha_{D}$($\beta_{L}\alpha_{D}$) | $\beta_{L}\delta_{L}$($\beta_{L}\gamma_{L}$) | $\beta_{L}\gamma_{L}$($\beta_{L}\gamma_{L}$) | $\varepsilon_{L}\alpha_{D}$($\varepsilon_{L}\alpha_{D}$) | $\varepsilon_{L}\delta_{L}$($\varepsilon_{L}\gamma_{L}$) | $\varepsilon_{L}\gamma_{L}$($\varepsilon_{L}\gamma_{L}$) |
| $\alpha_{D}\gamma_{D}$($\alpha_{D}\gamma_{D}$) | $\alpha_{D}\delta_{D}$($\alpha_{D}\alpha_{D}$) | $\alpha_{D}\alpha_{L}$($\alpha_{D}\gamma_{L}$) | $\delta_{L}\gamma_{D}$($\beta_{L}\gamma_{D}$) | $\delta_{L}\delta_{D}$($\beta_{L}\delta_{D}$) | $\delta_{L}\alpha_{L}$($\delta_{L}\gamma_{L}$) | $\gamma_{L}\gamma_{D}$($\gamma_{L}\gamma_{D}$) | $\gamma_{L}\delta_{D}$($\gamma_{L}\delta_{D}$) | $\gamma_{L}\alpha_{L}$($\gamma_{L}\gamma_{L}$) |
| $\alpha_{D}\varepsilon_{D}$($\alpha_{D}\gamma_{D}$) | $\alpha_{D}\beta_{L}$($\alpha_{D}\beta_{L}$) | $\alpha_{D}\varepsilon_{L}$($\delta_{D}\beta_{L}^{*}$) | $\delta_{L}\varepsilon_{D}$($\delta_{L}\varepsilon_{D}$) | $\delta_{L}\beta_{L}$($\delta_{L}\beta_{L}$) | $\delta_{L}\varepsilon_{L}$($\delta_{L}\beta_{L}$) | $\gamma_{L}\varepsilon_{D}$($\gamma_{L}\varepsilon_{D}$) | $\gamma_{L}\beta_{L}$($\gamma_{L}\beta_{L}$) | $\gamma_{L}\varepsilon_{L}$($\gamma_{L}\varepsilon_{L}$) |
| $\alpha_{D}\alpha_{D}$($\alpha_{D}\alpha_{D}$) | $\alpha_{D}\delta_{L}$($\alpha_{D}\gamma_{L}$) | $\alpha_{D}\gamma_{L}$($\alpha_{D}\gamma_{L}$) | $\delta_{L}\alpha_{D}$($\beta_{L}\gamma_{D}^{*}$) | $\delta_{L}\delta_{L}$($\delta_{L}\gamma_{L}$) | $\delta_{L}\gamma_{L}$($\delta_{L}\gamma_{L}$) | $\gamma_{L}\alpha_{D}$($\gamma_{L}\alpha_{D}$) | $\gamma_{L}\delta_{L}$($\gamma_{L}\gamma_{L}$) | $\gamma_{L}\gamma_{L}$($\gamma_{L}\gamma_{L}$) |
| ***gauche (+)*** | | | | | | | | |
| $\gamma_{D}\gamma_{D}$($\gamma_{D}\gamma_{D}$) | $\gamma_{D}\delta_{D}$($\varepsilon_{D}\gamma_{L}^{*}$) | $\gamma_{D}\alpha_{L}$($\gamma_{D}\alpha_{L}$) | $\delta_{D}\gamma_{D}$($\alpha_{D}\gamma_{D}$) | $\delta_{D}\delta_{D}$($\alpha_{D}\delta_{D}$) | $\delta_{D}\alpha_{L}$($\gamma_{D}\gamma_{L}^{*}$) | $\alpha_{L}\gamma_{D}$($\alpha_{L}\gamma_{D}$) | $\alpha_{L}\delta_{D}$($\alpha_{L}\delta_{D}$) | $\alpha_{L}\alpha_{L}$($\alpha_{L}\gamma_{L}$) |
| $\gamma_{D}\varepsilon_{D}$($\gamma_{D}\gamma_{D}$) | $\gamma_{D}\beta_{L}$($\gamma_{D}\beta_{L}$) | $\gamma_{D}\varepsilon_{L}$($\gamma_{D}\gamma_{L}$) | $\delta_{D}\varepsilon_{D}$($\alpha_{D}\gamma_{D}^{*}$) | $\delta_{D}\beta_{L}$($\gamma_{D}\beta_{L}$) | $\delta_{D}\varepsilon_{L}$($\gamma_{D}\beta_{L}^{*}$) | $\alpha_{L}\varepsilon_{D}$($\alpha_{L}\gamma_{D}$) | $\alpha_{L}\beta_{L}$($\alpha_{L}\beta_{L}$) | $\alpha_{L}\varepsilon_{L}$($\alpha_{L}\beta_{L}$) |
| $\gamma_{D}\alpha_{D}$($\gamma_{D}\alpha_{D}$) | $\gamma_{D}\delta_{L}$($\varepsilon_{D}\gamma_{L}^{*}$) | $\gamma_{D}\gamma_{L}$($\gamma_{D}\gamma_{L}$) | $\delta_{D}\alpha_{D}$($\alpha_{D}\alpha_{D}$) | $\delta_{D}\delta_{L}$($\alpha_{D}\delta_{L}$) | $\delta_{D}\gamma_{L}$($\alpha_{D}\gamma_{L}$) | $\alpha_{L}\alpha_{D}$($\alpha_{L}\alpha_{D}$) | $\alpha_{L}\delta_{L}$($\alpha_{L}\gamma_{L}$) | $\alpha_{L}\gamma_{L}$($\alpha_{L}\gamma_{L}$) |
| $\varepsilon_{D}\gamma_{D}$($\gamma_{D}\gamma_{D}$) | $\varepsilon_{D}\delta_{D}$($\varepsilon_{D}\gamma_{L}$) | $\varepsilon_{D}\alpha_{L}$($\varepsilon_{D}\gamma_{L}$) | $\beta_{L}\gamma_{D}$($\gamma_{D}\gamma_{D}$) | $\beta_{L}\delta_{D}$($\beta_{L}\delta_{D}$) | $\beta_{L}\alpha_{L}$($\varepsilon_{D}\gamma_{L}^{*}$) | $\varepsilon_{L}\gamma_{D}$($\gamma_{L}\gamma_{D}$) | $\varepsilon_{L}\delta_{D}$($\gamma_{L}\delta_{D}$) | $\varepsilon_{L}\alpha_{L}$($\gamma_{L}\delta_{L}^{*}$) |
| $\varepsilon_{D}\varepsilon_{D}$($\gamma_{D}\gamma_{D}^{*}$) | $\varepsilon_{D}\beta_{L}$($\varepsilon_{D}\beta_{L}$) | $\varepsilon_{D}\varepsilon_{L}$($\varepsilon_{D}\beta_{L}$) | $\beta_{L}\varepsilon_{D}$($\beta_{L}\varepsilon_{D}$) | $\beta_{L}\beta_{L}$($\beta_{L}\beta_{L}$) | $\beta_{L}\varepsilon_{L}$($\gamma_{D}\gamma_{L}^{*}$) | $\varepsilon_{L}\varepsilon_{D}$($\gamma_{D}\gamma_{D}^{*}$) | $\varepsilon_{L}\beta_{L}$($\gamma_{L}\beta_{L}$) | $\varepsilon_{L}\varepsilon_{L}$($\gamma_{D}\gamma_{L}^{*}$) |
| $\varepsilon_{D}\alpha_{D}$($\gamma_{D}\alpha_{D}$) | $\varepsilon_{D}\delta_{L}$($\varepsilon_{D}\gamma_{L}$) | $\varepsilon_{D}\gamma_{L}$($\gamma_{D}\gamma_{L}$) | $\beta_{L}\alpha_{D}$($\gamma_{D}\alpha_{D}$) | $\beta_{L}\delta_{L}$($\varepsilon_{D}\gamma_{L}^{*}$) | $\beta_{L}\gamma_{L}$($\gamma_{D}\gamma_{L}$) | $\varepsilon_{L}\alpha_{D}$($\varepsilon_{L}\alpha_{D}$) | $\varepsilon_{L}\delta_{L}$($\gamma_{L}\delta_{L}$) | $\varepsilon_{L}\gamma_{L}$($\gamma_{L}\gamma_{L}$) |
| $\alpha_{D}\gamma_{D}$($\alpha_{D}\gamma_{D}$) | $\alpha_{D}\delta_{D}$($\alpha_{D}\alpha_{D}$) | $\alpha_{D}\alpha_{L}$($\gamma_{D}\gamma_{L}^{*}$) | $\delta_{L}\gamma_{D}$($\alpha_{D}\gamma_{D}$) | $\delta_{L}\delta_{D}$($\alpha_{D}\alpha_{D}^{*}$) | $\delta_{L}\alpha_{L}$($\alpha_{D}\gamma_{L}^{*}$) | $\gamma_{L}\gamma_{D}$($\gamma_{L}\gamma_{D}$) | $\gamma_{L}\delta_{D}$($\gamma_{L}\delta_{D}$) | $\gamma_{L}\alpha_{L}$($\gamma_{L}\delta_{L}$) |
| $\alpha_{D}\varepsilon_{D}$($\alpha_{D}\gamma_{D}$) | $\alpha_{D}\beta_{L}$($\alpha_{D}\beta_{L}$) | $\alpha_{D}\varepsilon_{L}$($\alpha_{D}\gamma_{L}$) | $\delta_{L}\varepsilon_{D}$($\delta_{L}\varepsilon_{D}$) | $\delta_{L}\beta_{L}$($\alpha_{D}\beta_{L}$) | $\delta_{L}\varepsilon_{L}$($\gamma_{D}\beta_{L}^{*}$) | $\gamma_{L}\varepsilon_{D}$($\gamma_{L}\gamma_{D}$) | $\gamma_{L}\beta_{L}$($\gamma_{L}\beta_{L}$) | $\gamma_{L}\varepsilon_{L}$($\gamma_{L}\gamma_{L}$) |
| $\alpha_{D}\alpha_{D}$($\alpha_{D}\alpha_{D}$) | $\alpha_{D}\delta_{L}$($\alpha_{D}\delta_{L}$) | $\alpha_{D}\gamma_{L}$($\alpha_{D}\gamma_{L}$) | $\delta_{L}\alpha_{D}$($\alpha_{D}\gamma_{D}^{*}$) | $\delta_{L}\delta_{L}$($\alpha_{D}\gamma_{L}^{*}$) | $\delta_{L}\gamma_{L}$($\alpha_{D}\gamma_{L}$) | $\gamma_{L}\alpha_{D}$($\gamma_{L}\alpha_{D}$) | $\gamma_{L}\delta_{L}$($\gamma_{L}\delta_{L}$) | $\gamma_{L}\gamma_{L}$($\gamma_{L}\gamma_{L}$) |

**Table S1** continued.

| ***gauche (-)*** | | | | | | | | |
| --- | --- | --- | --- | --- | --- | --- | --- | --- |
| $\gamma_{D}\gamma_{D}$($\gamma_{D}\gamma_{D}$) | $\gamma_{D}\delta_{D}$($\gamma_{D}\gamma_{L}$) | $\gamma_{D}\alpha_{L}$($\gamma_{D}\gamma_{L}$) | $\delta_{D}\gamma_{D}$($\gamma_{D}\gamma_{D}$) | $\delta_{D}\delta_{D}$($\gamma_{D}\delta_{D}$) | $\delta_{D}\alpha_{L}$($\alpha_{D}\gamma_{L}^{*}$) | $\alpha_{L}\gamma_{D}$($\alpha_{L}\gamma_{D}$) | $\alpha_{L}\delta_{D}$($\gamma_{L}\alpha_{L}^{*}$) | $\alpha_{L}\alpha_{L}$($\alpha_{L}\gamma_{L}$) |
| $\gamma_{D}\varepsilon_{D}$($\gamma_{D}\gamma_{D}$) | $\gamma_{D}\beta_{L}$($\gamma_{D}\gamma_{L}$) | $\gamma_{D}\varepsilon_{L}$($\gamma_{D}\gamma_{L}$) | $\delta_{D}\varepsilon_{D}$($\gamma_{D}\gamma_{D}^{*}$) | $\delta_{D}\beta_{L}$($\gamma_{D}\beta_{L}$) | $\delta_{D}\varepsilon_{L}$($\gamma_{D}\beta_{L}^{*}$) | $\alpha_{L}\varepsilon_{D}$($\alpha_{L}\gamma_{D}$) | $\alpha_{L}\beta_{L}$($\alpha_{L}\beta_{L}$) | $\alpha_{L}\varepsilon_{L}$($\gamma_{L}\gamma_{L}^{*}$) |
| $\gamma_{D}\alpha_{D}$($\gamma_{D}\alpha_{D}$) | $\gamma_{D}\delta_{L}$($\gamma_{D}\gamma_{L}$) | $\gamma_{D}\gamma_{L}$($\gamma_{D}\gamma_{L}$) | $\delta_{D}\alpha_{D}$($\alpha_{D}\alpha_{D}$) | $\delta_{D}\delta_{L}$($\delta_{L}\gamma_{L}^{*}$) | $\delta_{D}\gamma_{L}$($\gamma_{D}\gamma_{L}$) | $\alpha_{L}\alpha_{D}$($\gamma_{L}\alpha_{D}$) | $\alpha_{L}\delta_{L}$($\alpha_{L}\gamma_{L}$) | $\alpha_{L}\gamma_{L}$($\alpha_{L}\gamma_{L}$) |
| $\varepsilon_{D}\gamma_{D}$($\delta_{L}\gamma_{D}$) | $\varepsilon_{D}\delta_{D}$($\beta_{L}\delta_{D}$) | $\varepsilon_{D}\alpha_{L}$($\varepsilon_{D}\gamma_{L}$) | $\beta_{L}\gamma_{D}$($\beta_{L}\gamma_{D}$) | $\beta_{L}\delta_{D}$($\beta_{L}\delta_{D}$) | $\beta_{L}\alpha_{L}$($\beta_{L}\delta_{L}$) | $\varepsilon_{L}\gamma_{D}$($\gamma_{L}\gamma_{D}$) | $\varepsilon_{L}\delta_{D}$($\gamma_{L}\alpha_{D}^{*}$) | $\varepsilon_{L}\alpha_{L}$($\gamma_{L}\delta_{L}^{*}$) |
| $\varepsilon_{D}\varepsilon_{D}$($\beta_{L}\varepsilon_{D}$) | $\varepsilon_{D}\beta_{L}$($\beta_{L}\beta_{L}$) | $\varepsilon_{D}\varepsilon_{L}$($\beta_{L}\beta_{L}^{*}$) | $\beta_{L}\varepsilon_{D}$($\beta_{L}\varepsilon_{D}$) | $\beta_{L}\beta_{L}$($\beta_{L}\beta_{L}$) | $\beta_{L}\varepsilon_{L}$($\beta_{L}\beta_{L}$) | $\varepsilon_{L}\varepsilon_{D}$($\gamma_{L}\gamma_{D}^{*}$) | $\varepsilon_{L}\beta_{L}$($\gamma_{L}\beta_{L}$) | $\varepsilon_{L}\varepsilon_{L}$($\gamma_{L}\gamma_{L}^{*}$) |
| $\varepsilon_{D}\alpha_{D}$($\beta_{L}\alpha_{D}$) | $\varepsilon_{D}\delta_{L}$($\varepsilon_{D}\gamma_{L}$) | $\varepsilon_{D}\gamma_{L}$($\beta_{L}\gamma_{L}$) | $\beta_{L}\alpha_{D}$($\beta_{L}\alpha_{D}$) | $\beta_{L}\delta_{L}$($\beta_{L}\gamma_{L}$) | $\beta_{L}\gamma_{L}$($\beta_{L}\gamma_{L}$) | $\varepsilon_{L}\alpha_{D}$($\gamma_{L}\alpha_{D}$) | $\varepsilon_{L}\delta_{L}$($\gamma_{L}\delta_{L}$) | $\varepsilon_{L}\gamma_{L}$($\gamma_{L}\gamma_{L}$) |
| $\alpha_{D}\gamma_{D}$($\gamma_{D}\gamma_{D}$) | $\alpha_{D}\delta_{D}$($\gamma_{D}\delta_{D}$) | $\alpha_{D}\alpha_{L}$($\alpha_{D}\gamma_{L}$) | $\delta_{L}\gamma_{D}$($\delta_{L}\gamma_{D}$) | $\delta_{L}\delta_{D}$($\delta_{L}\delta_{D}$) | $\delta_{L}\alpha_{L}$($\delta_{L}\gamma_{L}$) | $\gamma_{L}\gamma_{D}$($\gamma_{L}\gamma_{D}$) | $\gamma_{L}\delta_{D}$($\gamma_{L}\delta_{D}$) | $\gamma_{L}\alpha_{L}$($\gamma_{L}\gamma_{L}$) |
| $\alpha_{D}\varepsilon_{D}$($\delta_{L}\gamma_{D}^{*}$) | $\alpha_{D}\beta_{L}$($\gamma_{D}\beta_{L}$) | $\alpha_{D}\varepsilon_{L}$($\alpha_{D}\varepsilon_{L}$) | $\delta_{L}\varepsilon_{D}$($\delta_{L}\varepsilon_{D}$) | $\delta_{L}\beta_{L}$($\gamma_{L}\beta_{L}$) | $\delta_{L}\varepsilon_{L}$($\alpha_{D}\varepsilon_{L}$) | $\gamma_{L}\varepsilon_{D}$($\gamma_{L}\gamma_{D}$) | $\gamma_{L}\beta_{L}$($\gamma_{L}\beta_{L}$) | $\gamma_{L}\varepsilon_{L}$($\gamma_{L}\varepsilon_{L}$) |
| $\alpha_{D}\alpha_{D}$($\alpha_{D}\alpha_{D}$) | $\alpha_{D}\delta_{L}$($\alpha_{D}\gamma_{L}$) | $\alpha_{D}\gamma_{L}$($\gamma_{D}\gamma_{L}$) | $\delta_{L}\alpha_{D}$($\alpha_{D}\alpha_{D}$) | $\delta_{L}\delta_{L}$($\gamma_{L}\gamma_{L}^{*}$) | $\delta_{L}\gamma_{L}$($\delta_{L}\gamma_{L}$) | $\gamma_{L}\alpha_{D}$($\gamma_{L}\alpha_{D}$) | $\gamma_{L}\delta_{L}$($\gamma_{L}\gamma_{L}$) | $\gamma_{L}\gamma_{L}$($\gamma_{L}\gamma_{L}$) |

Eighty‒seven intrinsically stable conformations were found, 156 ones migrated to the different conformations of greater stability (in 36 conformers, (red ones as marked*), both the serine and alanine residues had migrated to the different conformations, and in 120 cases (blue ones) serine or alanine residue were migrated to the different conformations).

**Table S2.** Classiﬁcation of *β*−turn conformers of sa-protected dipeptide according to their type, torsional dihedrals**,$\psi_{i+1}$,** ,$\psi_{i+2}$ and dipole moment (*μ)* (Debye), at the B3LYP‒D3 and M06‒2X/6‒311+G (*d,p*) levels of theory in the gas and water phases.

| ***β*-turn type** | **Conformer** | **B3LYP-D3** | | | | **M06-2X (gas phase)** | | | |  | **M06-2X (water)** | | | |  |
| --- | --- | --- | --- | --- | --- | --- | --- | --- | --- | --- | --- | --- | --- | --- | --- |
|  |  |  | $\psi_{i+1}$ |  | $\psi_{i+2}$ |  | $\psi_{i+1}$ |  | $\psi_{i+2}$ | ***μ*** |  |  |  |  | ***μ*** |
| *I* | $\alpha_{L}^{a}\gamma_{L}$ | -58.07 | -40.14 | -84.21 | 76.79 | -55.82 | -42.24 | -83.89 | 76.76 | 4.79 | -58.03 | -38.26 | -85.98 | 69.62 | 7.22 |
|  | $\alpha_{L}^{+}\gamma_{L}$ | -60.55 | -38.46 | -83.39 | 75.92 | -57.83 | -41.36 | -83.08 | 76.66 | 5.19 | -60.83 | -35.25 | -84.92 | 70.35 | 6.96 |
|  | $\alpha_{L}^{-}\gamma_{L}$ | -46.26 | -54.82 | -83.71 | 79.3 | -45.59 | -54.47 | -82.47 | 81.24 | 5.60 | -44.01 | -51.90 | -82.34 | 77.66 | 8.45 |
| *I'* | $\alpha_{D}^{a}\gamma_{D}$ | 65.34 | 40.89 | 73.33 | -58.9 | 59.76 | 44.71 | 76.03 | -54.07 | 8.20 | 62.35 | 41.17 | 76.49 | -48.80 | 11.24 |
|  | $\alpha_{D}^{+}\gamma_{D}$ | 76.64 | 18.32 | 72.86 | -57.92 | 72.19 | 22.26 | 74.39 | -55.45 | 7.16 | 68.64 | 27.73 | 75.22 | -48.97 | 9.72 |
| *II* | $\varepsilon_{L}^{a}\gamma_{D}$ | -67.95 | 170.72 | 74.00 | -57.49 | -63.17 | 170.55 | 76.23 | -49.86 | 5.66 | -57.80 | 156.99 | 76.64 | -45.47 | 9.17 |
|  | $\varepsilon_{L}^{a}\delta_{D}$ | -67.35 | 159.95 | -168.06 | -45.05 | -62.60 | 160.44 | -170.61 | -39.13 | 5.14 | -58.38 | 154.15 | -165.13 | -42.15 | 9.00 |
| *III'* | $\alpha_{D}^{a}\alpha_{D}$ | 56.19 | 39.41 | 66.51 | 16.12 | 57.34 | 36.74 | 61.32 | 23.22 | 10.19 | 55.87 | 37.49 | 59.74 | 27.31 | 14.54 |
|  | $\alpha_{D}^{+}\alpha_{D}$ | 64.90 | 23.29 | 65.13 | 21.61 | 65.04 | 23.49 | 61.06 | 25.69 | 8.65 | 61.50 | 28.27 | 59.00 | 28.99 | 12.15 |
|  | $\alpha_{D}^{-}\alpha_{D}$ | 71.94 | 10.47 | 66.18 | 22.61 | 68.82 | 13.59 | 62.41 | 26.49 | 8.80 | 64.84 | 21.74 | 59.77 | 28.68 | 12.63 |
| *V* | $\gamma_{L}^{a}\gamma_{D}$ | -73.75 | 68.72 | 73.24 | -54.29 | -74.91 | 70.52 | 75.37 | -50.66 | 5.51 | -74.10 | 64.63 | 75.81 | -50.17 | 7.78 |
|  | $\gamma_{L}^{+}\gamma_{D}$ | -74.64 | 56.55 | 72.63 | -55.58 | -78.56 | 54.99 | 74.30 | -52.33 | 5.01 | -76.78 | 57.08 | 75.93 | -53.14 | 6.07 |
|  | $\gamma_{L}^{-}\gamma_{D}$ | -56.87 | 41.45 | 73.71 | -54.00 | -59.36 | 41.95 | 75.89 | -52.71 | 7.53 | -58.79 | 37.94 | 76.47 | -52.27 | 9.96 |
| *V’* | $\gamma_{D}^{a}\gamma_{L}$ | 82.24 | -66.80 | -81.97 | 71.97 | 84.19 | -65.77 | -83.44 | 68.75 | 3.64 | 84.97 | -64.01 | -84.99 | 68.37 | 5.15 |
|  | $\gamma_{D}^{+}\gamma_{L}$ | 82.49 | -67.41 | -81.43 | 71.57 | 84.08 | -64.66 | -83.13 | 69.83 | 5.67 | 85.14 | -68.82 | -84.62 | 67.69 | 7.14 |
|  | $\gamma_{D}^{-}\gamma_{L}$ | 81.88 | -72.81 | -81.77 | 71.64 | 83.11 | -71.81 | -83.28 | 68.16 | 3.1 | 84.18 | -71.85 | -84.91 | 68.57 | 4.27 |
| *VIa1* | $\varepsilon_{L}^{a}\gamma_{L}$ | -68.27 | 164.17 | -83.63 | 72.04 | -63.47 | 164.11 | -84.65 | 70.32 | 4.26 | -59.00 | 157.12 | -85.65 | 66.51 | 7.24 |
| *VIa2* | $\beta_{L}^{a}\gamma_{L}$ | 159.69 | 174.53 | -84.69 | 71.43 | 160.62 | 174.70 | -85.46 | 68.92 | 3.5 | 158.12 | 176.36 | -86.68 | 66.68 | 5.43 |
|  | $\beta_{L}^{-}\gamma_{L}$ | 161.12 | -173.56 | -85.67 | 73.54 | 163.55 | -175.31 | -86.72 | 70.71 | 2.7 | 159.03 | -169.49 | -86.94 | 66.42 | 3.3 |
| *VIII* | $\alpha_{L}^{a}\beta_{L}$ | -59.76 | -32.70 | -154.48 | 166.87 | -56.63 | -36.03 | -154.76 | 171.40 | 4.16 | -56.63 | -37.39 | -157.28 | 167.92 | 6.12 |
|  | $\alpha_{L}^{+}\beta_{L}$ | -62.34 | -32.03 | -154.19 | 166.00 | -59.31 | -34.76 | -154.06 | 171.99 | 1.15 | -59.34 | -35.20 | -155.09 | 167.57 | 3.25 |
|  | $\alpha_{L}^{-}\beta_{L}$ | -43.53 | -51.24 | -150.14 | 168.5 | -42.89 | -51.33 | -149.27 | 173.17 | 3.33 | -42.52 | -51.94 | -152.13 | 168.35 | 6.08 |

1. *Corresponding author. Tel.: +989155138349; Tel-fax.: +982332394289

   E-mail address: [bchahkandi@gmail.com](mailto:bchahkandi@gmail.com) (B.Chahkandi). [↑](#footnote-ref-1)
